# Shattering the “golden armor”: A natural supramolecular nanoplatform for optical virulence modulation and MRSA keratitis therapy

**DOI:** 10.1016/j.mtbio.2026.103374

**Published:** 2026-06-22

**Authors:** Zhi-heng Yang, Hong-miao Dang, Xiao Zhang, Ling-feng Xu, Lu-lu Wang, Xin Pang, You-hong Hu

**Affiliations:** a Department of Pharmacy, The First Affiliated Hospital of Zhengzhou University, Zhengzhou, Henan, 450052, China; b School of Pharmacy, Henan University of Chinese Medicine, Zhengzhou, 450046, China; c College of Chemical Engineering, Fuzhou University, Fujian, 350108, China

**Keywords:** Optical intervention, Photodynamic therapy, Staphyloxanthin, Methicillin-resistant *Staphylococcus aureus* keratitis, Corneal penetration, Supramolecular co-assembly

## Abstract

Methicillin-resistant *Staphylococcus aureus* (MRSA) keratitis constitutes a formidable therapeutic impasse, driven by the convergence of the pathogen's intrinsic antioxidant “golden armor” (staphyloxanthin, STX) and the cornea's extrinsic physical barrier. Herein, we overcome these dual obstacles via carrier-free supramolecular nanotherapeutic (HB@GA), engineered through the co-assembly of the natural photosensitizer hypocrellin B (HB) and the bioactive glycyrrhizic acid (GA). This minimalist all-active design not only ensures 100% active payload but also enables deep corneal drug delivery via GA-enhanced membrane permeation. Upon 460 nm irradiation, the system executes a synchronous disarm-and-kill cascade, where the wavelength-specific photobleaching of STX optically strips the bacterial antioxidant defense, rendering the pathogen hypersensitive to concurrent reactive oxygen species storm generated by HB. Beyond potent sterilization, the bioactive GA component fundamentally reshapes the inflammatory microenvironment by inhibiting the HMGB1/TLR4 signaling axis, which shifts macrophage polarization from a pro-inflammatory M1 to a tissue-reparative M2 phenotype. In a murine keratitis model, the HB@GA nanoplatform combined with light treatment achieved rapid bacterial clearance and accelerated corneal reconstruction. By seamlessly integrating optical bio-intervention with bioactive supramolecular nanomedicine, this work establishes a promising “anti-infection and pro-repair” paradigm for addressing multidrug-resistant ocular pathologies.

## Graphical abstract

**Figure:**
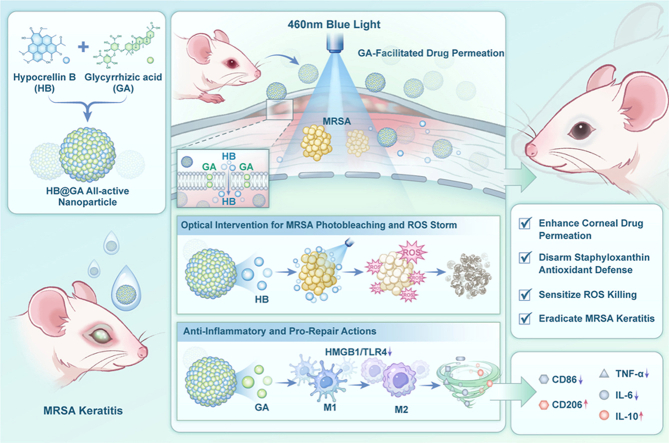

## 1 Introduction

The escalating crisis of multidrug-resistant (MDR) infections constitutes a dire global health burden, in which methicillin-resistant *Staphylococcus aureus* (MRSA)-induced keratitis represents a particularly formidable ophthalmic emergency [[1], [2], [3], [4]]. Characterized by rapid tissue melting and irreversible vision loss, the recalcitrance of this pathology stems from the pathogen's sophisticated survival mechanisms [3,5]. Central to its resilience is the intrinsic biochemical defense conferred by staphyloxanthin (STX), a carotenoid pigment functioning as a “golden armor” that efficiently quenches exogenous and host-derived reactive oxygen species (ROS) to subvert host immunity and oxidative therapeutics. Exacerbating this therapeutic dilemma is a formidable physical obstacle of dense corneal epithelium [6]. Its lipophilic nature and tight junctions create a rigorous anatomical barrier that severely restrict the transmucosal permeation of conventional antimicrobial agents [7], precluding effective drug accumulation at the infection site [8,9]. This distinct convergence of a biochemical antioxidant shield and a physio-anatomical barrier underscores the inadequacy of current treatments, necessitating the development of next-generation delivery systems engineered to simultaneously breach both defenses for effective intraocular biodistribution [10]. Recent advances have highlighted the potential of nanomaterial-based strategies for bacterial keratitis, including carbon quantum dots and carbonized nanogels, which exhibit intrinsic antibacterial and antioxidant activities [[1], [2], [3]]. Beyond these carbon-based nanomaterials, other functional nanoformulations such as immunomodulatory hydrogels, DNA supramolecular hydrogels, and hydrogen-generating nanofibrous membranes have demonstrated promising efficacy in promoting scarless wound healing and tissue regeneration, further supporting the versatility of nanotechnology for treating infectious diseases [[11], [12], [13], [14]].

Beyond traditional chemotherapy, optical-intervention strategies based on specific light-matter interactions offer a promising frontier [15]. Unlike optogenetics, this method exploits the native photosensitivity of bacterial chromophores without genetic manipulation. A prime example is the specific targeting of the STX pigment with 460 nm light. This wavelength functions as a “molecular switch”, triggering the rapid photolysis of the bacterium's “golden armor” and effectively dismantling its antioxidant defense system [16]. Such “optical disarming” not only sensitizes MRSA to oxidative attack but also opens a critical therapeutic window for treatments such as photodynamic therapy (PDT), which relies on light-activated generation of cytotoxic ROS [17]. In this context, Hypocrellin B (HB), a natural perylenequinone photosensitizer, emerges as an ideal candidate. Its high ROS yield is complemented by an absorption maximum that aligns seamlessly with the STX photobleaching peak at 460 nm. This spectral coincidence facilitates a “one-photon, dual-action” strategy, wherein a single wavelength simultaneously functions as a virulence attenuator (via STX degradation) and a PDT activator (via HB excitation), ensuring maximum bacterial lethality. Despite this theoretical elegance, the clinical translation of HB is fundamentally constrained by its extreme hydrophobicity and poor corneal permeability, which necessitates an advanced delivery strategy to unlock its full therapeutic potential [18].

Nature provides elegant answers to scientific conundrums, offering multifunctional building blocks for advanced therapeutics [[19], [20], [21], [22], [23]]. In this pursuit, *Glycyrrhiza glabra* (licorice), historically harnessed in traditional medicine for ocular inflammation, may serve as a sophisticated solution. Its principal bioactive constituent, glycyrrhizic acid (GA), exhibits unique physicochemical and immunomodulatory properties that directly counter the core obstacles in keratitis therapy. Structurally, GA functions as a natural amphiphile capable of intercalating into the corneal epithelial lipid bilayers. This interaction increases membrane fluidity and facilitates the transmembrane delivery of therapeutics, effectively “unlocking” the rigorous ocular barrier for hydrophobic cargos like HB. Biologically, GA orchestrates a critical tissue-repair mechanism [24,25]. By specifically inhibiting high mobility group box 1 (HMGB1) and blocking the downstream HMGB1/TLR4 signaling axis, GA drives the phenotypic reprogramming of macrophages from a pro-inflammatory M1 state to a reparative M2 state [26]. This immunomodulatory capability compensates for the cornea's intrinsic lack of regenerative potential [27]. Therefore, coupling GA with HB creates a conceptually novel nanoplatform where the carrier is also the therapeutic. In this system, GA functions as a solubilizer, enhancer, and immunomodulator, creating a powerful synergy with HB. This combination shifts the therapeutic paradigm from a simple “kill-only” approach to an integrated “eradicate-and-repair” strategy [28], ensuring both bacterial elimination and functional tissue recovery [29,30].

Inspired by the emerging paradigm of carrier-free nanomedicine [[31], [32], [33]], we herein designed a fully bioactive nanotherapeutic (denoted HB@GA) via the supramolecular co-assembly of HB and GA. This minimalist design eliminates inert excipients, achieving a 100% therapeutic payload while streamlining the preparation process.

**Fig. 1:**
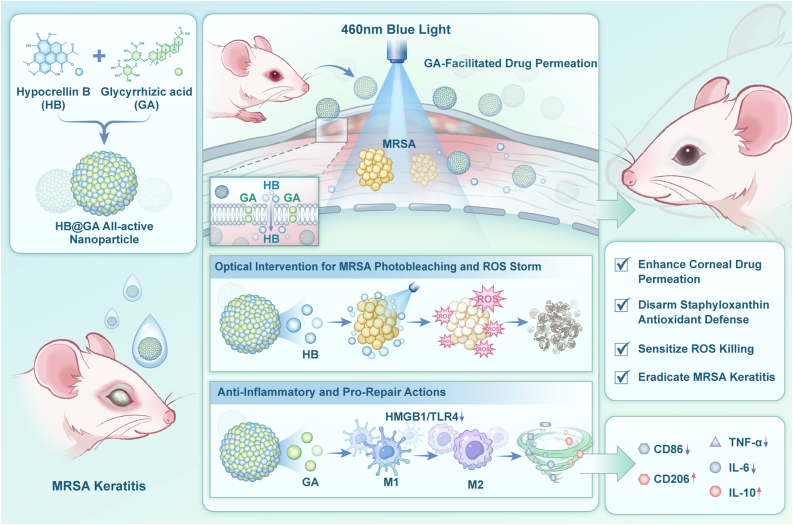
Schematic illustration of the fabrication and mechanism of HB@GA against bacterial keratitis.

Capitalizing on these insights, we engineered a fully bioactive, supramolecular co-assembled nanotherapeutic (denoted as HB@GA) guided by the philosophy of “carrier-free” nanomedicine [34]. By leveraging GA as both a functional building block and a therapeutic agent, this system eschews inert excipients to achieve a 100% active payload via facile self-assembly. The resulting HB@GA nanoparticles effectively traverse the corneal epithelial barrier, ensuring deep tissue accumulation at the infection nidus. Under 460 nm irradiation, the system orchestrates a synchronous “disarm-and-kill” mechanism: the specific photobleaching of STX strips MRSA of its antioxidant “golden armor”, rendering the pathogen defenseless against the simultaneous HB-mediated ROS storm. Beyond bacterial eradication, the bioactive carrier GA actively drives corneal reconstruction. By inhibiting the HMGB1/TLR4 signaling axis, GA modulates macrophage polarization from the pro-inflammatory M1 to the reparative M2 phenotype. In murine models, this comprehensive treatment achieved the complete recovery of MRSA-induced keratitis (Fig. 1). Ultimately, this study validates a promising strategy that synergizes precise optical interference with natural supramolecular assembly, establishing a robust “anti-infection and pro-repair” paradigm to address the complexities of MDR ocular diseases.

## 2 Experimental section/methods

### 2.1 Preparation and characterization of the HB@GA

HB@GA nanoparticles were prepared using a solvent-antisolvent precipitation method. Specifically, 0.2 mg of hypocrellin B (HB) was accurately weighed and dissolved in 0.2 mL of ethanol under brief sonication (5 min) to obtain a 1 mg/mL stock solution (solvent phase). Simultaneously, 4 mg of glycyrrhizic acid (GA) was precisely weighed and dissolved in 1 mL of deionized water to prepare a 4 mg/mL solution (antisolvent phase). The HB solution (solvent phase) was then slowly injected into the GA solution (antisolvent phase) under continuous magnetic stirring (1000 rpm, 25°C) with protection from light. The mixture was stirred for an additional 24 h under light-shielded conditions. Subsequently, the system was centrifuged at 12,000 rpm for 15 min. The supernatant was carefully removed, and the resulting pellet was washed twice with deionized water. Finally, the purified nanoparticles were resuspended in phosphate-buffered saline (PBS) and stored at 4°C until further use.

The size distribution and polydispersity index (PDI) of the HB@GA were determined using a Zetasizer (90Plus PALS, NanoBrook). The morphology was investigated via SEM (ZEISS, Gemini 300) operating at 200 kV acceleration voltage. For SEM sample preparation, an aqueous suspension of HB@GA was placed onto a copper grid and allowed to dry at ambient temperature. Additionally, the UV/Vis absorption spectra and fluorescence spectra were recorded by employing a microplate reader (SpectraMax iD5, Molecular Devices). Fourier transform infrared spectroscopy (FTIR, Thermo Fisher Scientific, Waltham, MA, USA) was used to obtain the spectrum of HB, GA and HB@GA in the wave number region of 500−4000 cm-1.

### 2.2 Reactive oxygen species (ROS) production of HB@GA

Reactive oxygen species (ROS) generation by HB@GA was quantified via DCFH-DA fluorescence assay. Specifically, gradient concentrations of HB@GA solutions were incubated with 10 μM DCFH-DA for 30 min. Following this, the mixtures were irradiated with 460 nm laser light (100 mW/cm^2^) for 5 min. Fluorescence intensity of oxidized DCF was then measured at 488 nm excitation and 400-550 nm emission to quantify ROS yield.

### 2.3 Drug releasing of HB@GA

Drug release profiling of HB@GA nanoparticles was conducted under physiologically relevant pH conditions. Specifically: pH 7.4 phosphate-buffered saline (PBS) simulated physiological environments and pH 5.0 PBS modeled acidic lysosomal conditions. Solid HB@GA nanoparticles were separately dispersed in 20 mL of pH 7.4 PBS containing 1% Tween-80 and pH 5.0 PBS containing 1% Tween-80. Samples were protected from light and incubated at 37°C in a thermostatic shaker (1500 rpm). Aliquots (600 μL) were collected at 0-48 h post-incubation, with immediate replenishment using equal volumes of fresh corresponding PBS. Fluorescence intensity spectra of collected samples were measured to quantify HB release kinetics from the nanocarrier under varying pH conditions.

### 2.4 Molecular dynamics simulation of HB@GA based supramolecular assemblies

To elucidate the molecular mechanism underlying the co-assembly of Hypocrellin B (HB) and Glycyrrhizic Acid (GA), all-atom molecular dynamics (MD) simulations were performed using the GROMACS-2019.6 software package [33]. The initial three-dimensional molecular structures of HB and GA were obtained from the PubChem database. The General Amber Force Field (GAFF) with AM1-BCC partial charges was employed to describe the intramolecular and intermolecular interactions. A cubic simulation box with dimensions of 10 nm × 10 nm × 10 nm was constructed, containing ten molecules each of HB and GA, solvated with the TIP3P explicit water model. To neutralize the system's net charge, an appropriate number of sodium counterions were added, replacing random water molecules. The solvated system subsequently underwent a standard equilibration protocol: first, energy minimization using the steepest descent algorithm to relieve steric clashes; second, a 100 ps canonical (NVT) ensemble simulation to stabilize the temperature at 298 K using the Nosé-Hoover thermostat; and third, a 100 ps isothermal-isobaric (NPT) ensemble simulation to equilibrate the density at 1.0 bar using the Parrinello-Rahman barostat. Finally, a production MD simulation was carried out under NPT conditions for 100 ns. Trajectory frames were saved every 10 ps for subsequent analysis. All visualizations and structural renderings were generated using Visual Molecular Dynamics (VMD) software, version 1.9.3 [35].

### 2.5 *In vitro* antibacterial activity assay of HB@GA

The MRSA bacteria were cultured in lysogeny broth (LB) medium under shaking at 37°C and harvested at the logarithmic growth phase prior to experiments. The concentration of bacteria was determined by measuring the optical density at 600 nm via UV-vis spectroscopy. MRSA suspensions were divided into following groups: PBS, H_2_O_2_, HB, and HB@GA; Each groups were subdivided into two condition: dark and laser. Briefly, the bacteria suspension with a final concentration of 10^6^ CFU mL^−1^ was evenly mixed with such material solutions at 25 μM of HB. The required samples were irradiated with 460 nm laser light (100 mW/cm^2^) for 10 min. Then, aliquots from the suspensions were gradually diluted, and 100 μL of diluted bacterial solutions was plated on LB agar plates. The number of bacterial colonies was recorded following an overnight culture at 37°C. Bacterial colonies of control group and bacteria treated with HB group were counted according to the procedure described above. Each sample was prepared in triplicate.

### 2.6 Live/dead bacterial staining assay

The bacteria suspensions from different groups were treated with a mixture of SYTO 9 and PI in the dark for 30 min at room temperature. After being washed three times with PBS, the samples (10 μL) were mounted on glass slides and observed using laser scanning confocal microscope (Nikon Ti2-A).

### 2.7 Intracellular ROS detection in bacteria

The ROS-generating capacity of HB@GA *in vitro* was validated using a DCFH-DA assay kit. Aliquots of uniformly cultured MRSA suspensions were allocated to experimental groups and incubated at 37°C for 6 h. During incubation, samples received light irradiation (100 mW/cm^2^, 10 min). Bacterial pellets were subsequently collected and incubated with 10 μM DCFH-DA under light-protected conditions at 37°C for 30 min. Following three wash cycles to remove excess probe, pellets were resuspended in 300 μL physiological saline. For imaging, 200 μL aliquots were transferred to confocal dishes and intracellular fluorescence was visualized using laser scanning confocal microscopy.

### 2.8 Morphology study of bacteria

To visualize the morphological alterations induced by treatment, bacterial cells from experimental groups were harvested via centrifugation (5000 × g, 10 min). The resulting pellets were fixed in 2.5% (v/v) glutaraldehyde for 4 h at 4°C. Following fixation, samples were dehydrated through a graded ethanol series (30%, 50%, 70%, 80%, 90%, and 100%, v/v), with a 15-min incubation at each concentration. The dehydrated specimens were then critically point-dried, sputter-coated with a thin layer of gold-palladium, and subsequently mounted on a silicon substrate for imaging. Ultrastructural analysis was performed using field-emission scanning electron microscopy (FE-SEM).

### 2.9 Quantification of bacterial DNA and protein leakage

DNA leakage assessment: Methicillin-resistant *Staphylococcus aureus* (MRSA) was cultured in liquid LB medium at 37°C until reaching mid-log phase (OD_600_ = 0.6). Bacterial cells were harvested by centrifugation (5000 rpm, 5 min), washed twice with phosphate-buffered saline (PBS), and resuspended to OD_600_ = 1.0. Following treatment with experimental antimicrobial agents for 4 h at 37°C, extracellular DNA content was quantified by measuring absorbance at 260 nm using a NanoDrop spectrophotometer.

Protein leakage assessment: Using identically prepared and treated bacterial suspensions, supernatant containing leaked proteins was collected post-centrifugation (5000 rpm, 5 min). Aliquots (50 μL) were mixed with 200 μL BCA working reagent and incubated at 37°C for 30 min. Absorbance at 562 nm was measured, with total protein quantified against a standard curve.

### 2.10 Anti-biofilm activity assay

To evaluate the anti-biofilm efficacy of HB@GA, MRSA was cultured to logarithmic phase and diluted to 1 × 10^6^ CFU/mL in fresh medium. One milliliter of the bacterial suspension was added to each well of a 48-well plate and incubated statically at 37°C for 24 h. The supernatant was then discarded, replaced with 1 mL of fresh medium, and incubated for another 24 h to obtain mature biofilms. After gentle washing with saline, the established biofilms were divided into six groups: (1) PBS, (2) PBS + 660 nm laser, (3) PBS + 460 nm laser, (4) HB@GA alone, (5) HB@GA + 660 nm laser, and (6) HB@GA + 460 nm laser. The samples were incubated for 6 h at 37°C, followed by laser irradiation where applicable. For crystal violet staining, the supernatant was removed, the biofilms were gently washed twice with saline, and 200 μL of 0.5 mg/mL crystal violet solution was added to each well. After incubation at 37°C for 4 h in the dark, the wells were photographed, washed with water, and the remaining crystal violet was dissolved in 1000 μL of absolute ethanol. The absorbance at 590 nm was measured using a microplate reader.

### 2.11 Live/dead staining of biofilms

Mature MRSA biofilms were prepared on removable Millicell EZ-Slide chambers. After the same treatment as described above, the slides were incubated with a staining mixture containing 1 μL each of SYTO 9 and propidium iodide (PI) diluted in 1000 μL of saline, for 30 min at 37°C in the dark. Unbound dye was removed by gentle washing with saline. The biofilms were then examined using a laser scanning confocal microscope with Z-stack acquisition and three-dimensional reconstruction to visualize structural integrity and bacterial viability.

### 2.12 Study of HMGB1-TLR4-mediated corneal epithelial-macrophage inflammatory response in a transwell co-culture system

Establishment of the co-culture system: A non-contact, bilayer transwell co-culture system was established to model the interaction between corneal epithelial cells and macrophages. RAW264.7 murine macrophages were cultured and maintained in Dulbecco's Modified Eagle Medium (DMEM) supplemented with 10% fetal bovine serum (FBS; obtained from Beisuo (Henan) Biotechnology Co., Ltd.). For the assay, macrophages were harvested using 0.25% trypsin-EDTA, counted, and seeded into the lower chambers of a 24-well plate at a density of 1 × 10^5^ cells per well in 1 mL of complete medium. The cells were allowed to adhere overnight under standard conditions (37°C, 5% CO_2_).Simultaneously, human corneal epithelial (HCE-T) cells were prepared. Transwell inserts (0.4 μm pore size, Corning) were pre-coated with 5 μg/mL fibronectin for 1 h at 37°C and washed twice with Dulbecco's phosphate-buffered saline (DPBS). HCE-T cells were detached using 0.05% trypsin-EDTA, counted, and seeded onto the apical side of the pre-coated inserts at a density of 2.5 × 10^4^ cells per insert in 0.3 mL of DMEM/F12 medium containing 10% FBS. The HCE-T monolayers were cultured for 72 h until a tight barrier was formed, confirmed by a transepithelial electrical resistance (TEER) value of ≥200 Ω cm^2^.

System assembly and experimental treatment: For the co-culture experiment, the HCE-T-seeded transwell inserts were placed into the 24-well plates containing the pre-seeded RAW264.7 macrophages. The medium was replaced with a low-serum assay medium (1% FBS) to minimize background cytokine interference: 0.3 mL of DMEM/F12 in the upper chamber and 1.0 mL of DMEM in the lower chamber.

In this system, the pro-inflammatory stimulus lipoteichoic acid (LTA) was added exclusively to the upper chamber. LTA stimulated HCE-T cells to release pro-inflammatory mediators, including HMGB1 and other cytokines. These factors subsequently diffused through the porous membrane and induced polarization of RAW264.7 macrophages in the lower chamber toward a pro-inflammatory M1 phenotype. This sequential process recapitulates the epithelial-macrophage crosstalk observed in bacterial keratitis.

The system was subjected to the following experimental treatments for 6 h (the peak time for HMGB1 release): Control Group: No LTA stimulation. LTA Group: LTA (from *Staphylococcus aureus*, 5 μg/mL) was added only to the upper chamber (HCE-T side). LTA + Glycyrrhizic Acid (GA) Group: LTA (5 μg/mL) and GA (10 μM) were co-administered to the upper chamber. LTA + HB Group: LTA (5 μg/mL) and free HB were co-administered to the upper chamber. LTA + HB@GA Group: LTA (5 μg/mL) and the HB@GA nanotherapeutic were co-administered to the upper chamber.

Sample collection and analysis: After the 6-h stimulation period, samples were collected for downstream analysis. The transwell inserts were carefully removed. The conditioned medium from the upper chamber (HCE-T side) was collected, centrifuged to remove cellular debris, and the supernatant was aliquoted and stored at −80°C for the quantification of HMGB1 released by epithelial cells. The medium from the lower chamber (macrophage side) was similarly collected, centrifuged, and the supernatant was stored at −80°C for the subsequent quantification of translocated HMGB1 and macrophage-derived cytokines (TNF-α, IL-6, IL-10) via enzyme-linked immunosorbent assay (ELISA). The macrophages in the lower chamber were fixed with 4% paraformaldehyde for subsequent immunofluorescence staining of intracellular targets.

### 2.13 The enhancing mechanisms of GA on the permeation of HB@GA NPs

To investigate the molecular mechanism of HB@GA nanoparticle permeation across the corneal epithelial barrier, all-atom molecular dynamics (MD) simulations were performed using the GROMACS 2019.6 software package [36]. A model of the corneal barrier was constructed as a hydrated phospholipid bilayer, designed to represent the apical membrane of corneal epithelial cells. The bilayer consisted of a mixture of 1-palmitoyl-2-oleoyl-sn-glycero-3-phosphocholine (POPC) and 1-palmitoyl-2-oleoyl-sn-glycero-3-phospho-L-serine (POPS) at a 7:3 M ratio, approximating the charge and physicochemical properties of a biological membrane. The simulation system was solvated with TIP3P water molecules in a periodic boundary box, and neutralized with an appropriate concentration of NaCl. Topology and parameters for all molecular components, including Hypocrellin B (HB), Glycyrrhizic Acid (GA), and the phospholipids, were assigned using the General AMBER Force Field (GAFF) with AM1-BCC charges. Long-range electrostatic interactions were treated using the Particle Mesh Ewald (PME) method. The system underwent sequential energy minimization, NVT equilibration, and NPT equilibration phases, followed by a production run of 100 ns under constant temperature (310 K) and pressure (1 bar) conditions, with trajectory frames saved every 10 ps for subsequent analysis.

### 2.14 *In vivo* corneal penetration study

The *in vivo* corneal penetration kinetics were assessed using a murine model. Healthy Balb/c mice were randomly assigned to experimental groups. A topical administration of HB@GA nano-suspension or HB solution was applied to the corneal surface of the right eye. At predetermined time intervals post-administration (30, 60, and 90 min), the animals were euthanized, and the treated eyeballs were immediately enucleated and rapidly frozen. Corneal sections with a thickness of 10 μm were obtained using a cryostat microtome. The distribution and relative intensity of the intrinsic red fluorescence of HB within the corneal layers (epithelium and stroma) were visualized and captured using a laser scanning confocal microscope under consistent imaging parameters (excitation/emission wavelengths, laser power, gain, and exposure time).

### 2.15 *In vivo* animal studies

Male Balb/c mice (6 weeks, 20 g) underwent intrastromal inoculation with 2 μL MRSA suspension (1 × 10^8^ CFU/mL). Successful infection was confirmed at 24 h post-inoculation (designated Day 0). Infected mice were randomized into eight treatment groups (n = 5): PBS, PBS + Laser, HB, HB + Laser, GA, GA + Laser, HB@GA and HB@GA + Laser.

Treatment protocol: Mice received daily topical ocular administration (10 μL/eye) of corresponding formulations at 25 μM HB-equivalent concentration, coupled with laser irradiation (460 nm, 50 mW/cm^2^, 30 min). Corneal pathology was documented via slit-lamp examination under white and cobalt blue light at Days 0, 1, 3, 5, and 7.

Terminal analysis (Day 7): Bacteriological assessment: Five mice per group were sacrificed. Eyeballs were homogenized in sterile PBS using an Ultra-Turrax homogenizer. Serial 10-fold dilutions were plated (100 μL) on LB agar for viable colony-forming unit (CFU) enumeration. Histopathological processing: Three mice per group were sacrificed. Eyeballs were enucleated and fixed in 4% paraformaldehyde. Tissues were dehydrated through a graded ethanol series (70%, 80%, 95%, 100%) and xylene (30 min per solution), then embedded in paraffin. Sections (4 μm thickness) were prepared for: Hematoxylin and eosin (H&E) staining, dual fluorescence staining with DAPI and FITC-conjugated MRSA probe and immunofluorescence analysis.

### 2.16 RNA sequencing analysis of corneal tissue

Total RNA was isolated from corneal tissue using the RNAprep Pure Tissue Kit (TIANGEN, DP441). Strand-specific mRNA sequencing libraries were prepared by ApexBio Technology LLC (Shanghai, China) with the Hieff NGS® Ultima Dual-mode mRNA Library Prep Kit (Yeasen, 12310 ES). Poly(A) + mRNA was enriched using oligo(dT) magnetic beads and reverse transcribed into cDNA. Sequencing was performed on an Illumina NovaSeq 6000 platform under a paired-end 150 bp configuration. Raw reads were processed with Trim Galore to remove adapter sequences and low-quality bases. Clean reads were aligned to the mouse reference genome (GRCm38/mm10) using HISAT2 [37]. Transcript assembly and quantification were conducted with StringTie [38]. Differentially expressed genes (DEGs) were identified using DESeq2 with the thresholds of |log2(fold change)| >1 and false discovery rate (FDR) < 0.05 [39]. Gene Ontology (GO) and KEGG pathway enrichment analyses were subsequently performed on the significant DEGs.

### 2.17 Biocompatibility assess of HB@GA *in vivo*

To evaluate the systemic and ocular safety profile of the HB@GA nanocomplex following repeated topical administration, an *in vivo* toxicity study was conducted. Male Balb/c mice (6 weeks old, approximately 20 g) were randomly allocated into three groups: a vehicle control group receiving phosphate-buffered saline (PBS), a laser-only group receiving PBS plus 460 nm laser irradiation, and a treatment group receiving the HB@GA formulation. Mice in all groups were administered a 10 μL topical dose (containing HB at a concentration of 25 μM for the HB@GA group, or PBS for the other groups) onto the corneal surface of each eye once daily for 7 consecutive days. The laser-only and HB@GA groups were exposed to 460 nm laser (50 mW/cm^2^, 30 min) immediately after each administration. Twenty-four hours after the final administration, all animals were humanely euthanized.

For histopathological assessment, major systemic organs, including the heart, liver, spleen, lungs, and kidneys, were harvested alongside the eyeballs. All tissues were fixed, processed, embedded in paraffin, and sectioned. Tissue sections were then stained with hematoxylin and eosin (H&E) and examined under an optical microscope for any morphological alterations or signs of toxicity. Whole blood samples were collected at the endpoint for complete blood count (CBC) using a standard automated hematology analyzer and for serum biochemistry using an automated clinical chemistry analyzer to assess liver and kidney function.

Additionally, serum levels of oxidative stress markers including malondialdehyde (MDA), superoxide dismutase (SOD), reduced glutathione (GSH), and catalase (CAT) were measured using commercial ELISA kits according to the manufacturer's protocols. Serum inflammatory cytokines (HMGB1, TNF-α, IL-6, and IL-10) were also quantified by ELISA. These analyses aimed to evaluate potential systemic oxidative stress and inflammatory responses induced by the treatments.

### 2.18 Statistical analysis

Data were displayed using mean ± SD deviation. The statistical significance was calculated using one-way analysis of variance (ANOVA) and two-way analysis of variance: ∗p < 0.05, ∗∗p < 0.01, ∗∗∗p < 0.001, ∗∗∗∗p < 0.0001.

## 3 Results and discussion

### 3.1 Preparation and characterization of the HB@GA nanotherapeutic

**Fig. 2:**
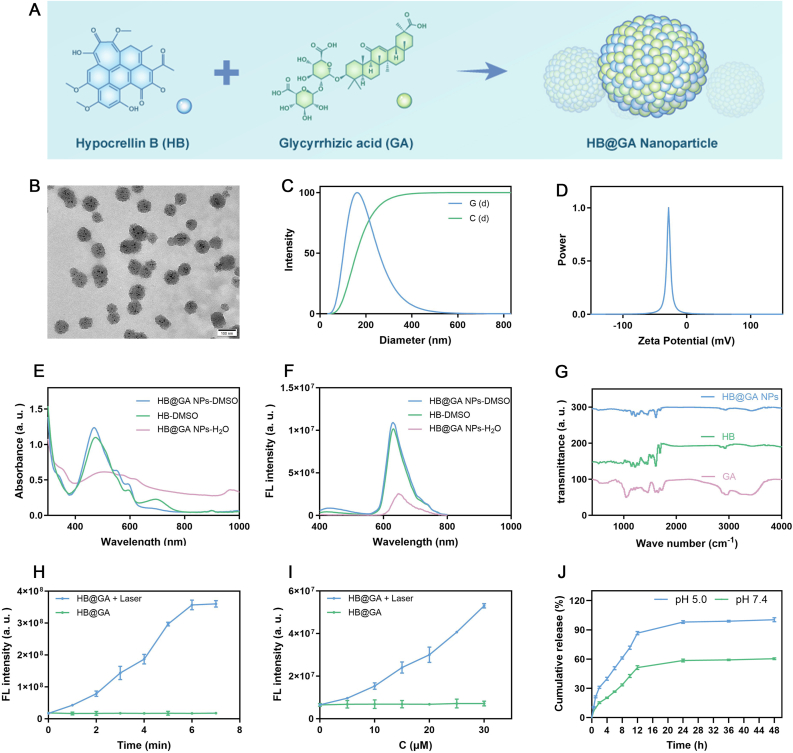
Characterization of HB@GA. (A) Schematic illustration of the co-assembly of HB@GA nanoparticles. (B) Transmission electron microscopy (TEM) image of the HB@GA. (C) Size distribution and (D) zeta potential of HB@GA in deionized water. (E) UV-vis-NIR absorption spectrum and (F) Fluorescence spectrum of HB@GA. (G) Infrared spectroscopy of HB, GA, HB@GA. DCF fluorescence intensity after HB@GA + Laser treatment at varying irradiation times (H) and concentrations (I). (J) Cumulative release profiles of HB at pH 5.0 and pH 7.4.

The carrier-free HB@GA nanotherapeutic was prepared through a facile solvent-antisolvent co-assembly strategy. Fig. 2A, illustrate the co-assembly process of HB@GA. Morphological analysis by transmission electron microscopy (TEM) revealed that the nanoparticles exhibited a uniform, quasi-spherical structure (Fig. 2B). This observation was quantitatively corroborated by dynamic light scattering (DLS), which measured an average hydrodynamic diameter of approximately 170 nm with a narrow polydispersity index (PDI), indicating a homogeneous size distribution (Fig. 2C). The colloidal stability of the freshly prepared nanosuspension was initially confirmed by a zeta potential of −29.7 mV (Fig. 2D), a value sufficiently negative to confer electrostatic repulsion and prevent aggregation. To ensure reliability under biologically relevant conditions, we next evaluated the long-term colloidal stability of HB@GA. As illustrated in Fig. S1, the nanoparticles retained their original size distribution over 7 days when incubated in phosphate-buffered saline (PBS), fetal bovine serum (FBS), and complete cell culture medium (DMEM). No significant increase in hydrodynamic diameter or visible aggregation was observed across these distinct media, highlighting the formulation's excellent serum stability and resistance to protein-induced aggregation. This robust stability profile is essential for maintaining consistent nanoparticle behavior throughout *in vitro* and *in vivo* studies, thereby ensuring dependable biodistribution and therapeutic performance.

Spectroscopic analysis was performed to investigate the intermolecular interactions underlying the co-assembly and the consequent photophysical properties of the HB@GA nanoplatform. The UV-Vis-NIR spectrum of free HB showed a characteristic π-π transition at 460 nm. After formulation into HB@GA nanoparticles, this absorption peak exhibited a noticeable red shift, accompanied by hypochromicity, band broadening, and baseline elevation (Fig. 2E). These spectral changes are consistent with the formation of molecular π-π stacks between HB molecules within the nanostructure, suggesting an ordered, densely packed arrangement in the nanoparticle core. Further insight into the local environment of HB was gained from fluorescence spectroscopy. HB@GA in aqueous buffer displayed an emission maximum at 660 nm. Its fluorescence intensity was significantly quenched compared to an equimolar solution of HB in dimethyl sulfoxide (DMSO) (Fig. 2F).

To identify the driving forces behind nanoparticle formation, we examined the UV-Vis absorption and fluorescence spectra of HB@GA in various chemical perturbants including urea, tetrahydrofuran (THF), sodium dodecyl sulfate (SDS), and sodium chloride (NaCl) (Figs. S2 and S3). Among these conditions, only THF, a solvent known to disrupt hydrophobic and π-π interactions, dramatically increased both the absorbance and fluorescence intensity of HB@GA to levels comparable to those of free HB in organic solvent. In contrast, urea (a hydrogen bond breaker), SDS (a chaotropic agent), and NaCl (an ionic disruptor) induced only minor spectral changes. These observations strongly indicate that hydrophobic forces and π-π stacking, rather than hydrogen bonding or electrostatic interactions, are the primary drivers for the co-assembly of HB and GA into stable nanoparticles. The successful incorporation of both bioactive components into a single supramolecular entity was confirmed by Fourier-transform infrared (FTIR) spectroscopy. The FTIR spectrum of HB@GA (Fig. 2G) retained the characteristic vibrational bands of both pristine HB and GA, confirming their coexistence without the formation of new covalent bonds. This evidence firmly supports a self-assembly process driven by non-covalent interactions, resulting in a supramolecular nanocomposite that preserves the chemical integrity of each constituent.

The core therapeutic function involving light-triggered reactive oxygen species generation was rigorously quantified using 2′,7′-dichlorofluorescin diacetate (DCFH-DA). This specific probe is initially non-fluorescent but converts into the highly fluorescent 2′,7′-dichlorofluorescein (DCF) upon oxidation by ROS. Upon irradiation with 460 nm light, the fluorescence intensity of DCF exhibited a continuous time-dependent increase that eventually reached a plateau after 6 min of exposure (Fig. 2H). Crucially, this ROS production was strictly dependent on light stimulation with negligible background signal observed in the dark. Furthermore, the system displayed a clear dose-response relationship regarding nanoparticle concentration as shown in Fig. 2I, which validates its utility for precise and controllable photodynamic therapy.

Finally, the drug release profile was evaluated under physiologically relevant conditions to mimic the transition from systemic circulation to the infectious microenvironment. Quantitative analysis over a 48-h period revealed a marked difference in HB release from HB@GA nanoparticles. At pH 5.0, mimicking the acidic infectious niche, the cumulative release reached approximately 98.6%. In contrast, under neutral physiological conditions (pH 7.4), only about 59.8% of HB was released (Fig. 2J). This pronounced pH-responsive release behavior is attributed to the protonation of the carboxyl groups in GA under acidic conditions. Such protonation weakens the supramolecular interactions holding the assembly together and triggers structural disassembly. This feature highlights an intelligent design capability for targeted drug activation specifically within the acidic milieu typical of bacterial infections.

### 3.2 Molecular dynamics simulation of HB@GA supramolecular assembly

**Fig. 3:**
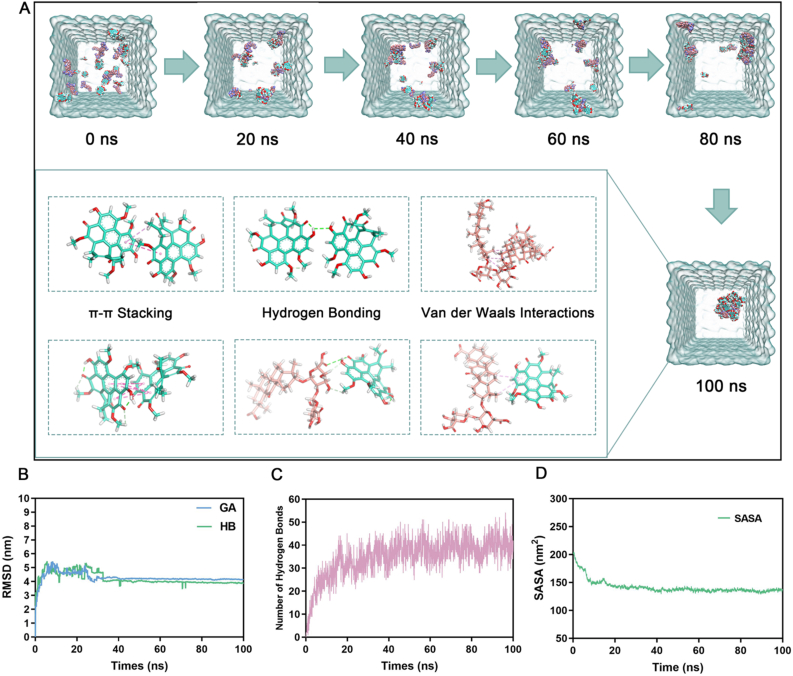
Molecular dynamic simulation of HB@GA. (A) The conformations and interactions of GA and HB at various time points throughout the 100 ns simulation. (B) RMSD plot of GA and HB showing structural stability of HB@GA during 100 ns simulation. (C) The number of hydrogen bonds formed between GA and HB during the simulation period. (D) Solvent Accessible Surface Area (SASA) plot of GA and HB during the simulation.

Molecular dynamics (MD) simulation provides critical atomistic insight into the dynamic evolution and intermolecular forces driving molecular self-assembly, complementing experimental observations [40,41]. To elucidate the co-assembly mechanism of HB and GA, an equimolar mixture of both molecules was initially dispersed randomly in a solvated periodic box. Over the course of the simulation, the system spontaneously transitioned from a disordered state to a well-defined, ordered aggregate (Fig. 3A). At the outset (0 ns), HB and GA molecules were uniformly distributed. Initial clustering became evident by 40 ns, progressed into distinct aggregates by 80 ns, and converged to a stable, fully assembled structure by approximately 100 ns. Trajectory analysis indicates that this co-assembly is governed by a synergistic interplay of π-π stacking, hydrogen bonding, and van der Waals interactions.

The structural stability of the resulting assembly was further quantified using root-mean-square deviation (RMSD) analysis (Fig. 3B). The RMSD curve showed initial variance corresponding to the rearrangement of monomers but settled into a steady plateau after 40 ns. This stabilization indicates that the system successfully reached an equilibrated state. To understand the binding mechanism, we tracked the number of intermolecular hydrogen bonds as shown in Fig. 3C. The results show a consistent presence of approximately 1.2 hydrogen bonds per pair in the equilibrated phase, highlighting the contribution of these interactions to the overall stability of the nanocomplex. Furthermore, the thermodynamic driver of this assembly was investigated by calculating the solvent-accessible surface area (Fig. 3D). A rapid decrease in SASA from ∼200 nm^2^ to ∼145 nm^2^ was observed within the first 20 ns. This reduction indicates the effective burial of hydrophobic surface areas during the assembly process. Subsequently, the number of hydrogen bonds remained relatively constant throughout the assembly process, confirming their critical role in stabilizing the self-assembled structure. Taken together, these results reveal that the supramolecular stability of HB@GA are governed by a synergistic interplay of π-π stacking, hydrogen bonding, and van der Waals interactions, which endow the system with robust self-assembly behavior in aqueous environments and lay a structural foundation for its subsequent biological applications.

### 3.3 *In vitro* antibacterial activity assay of HB@GA-based PDT

**Fig. 4:**
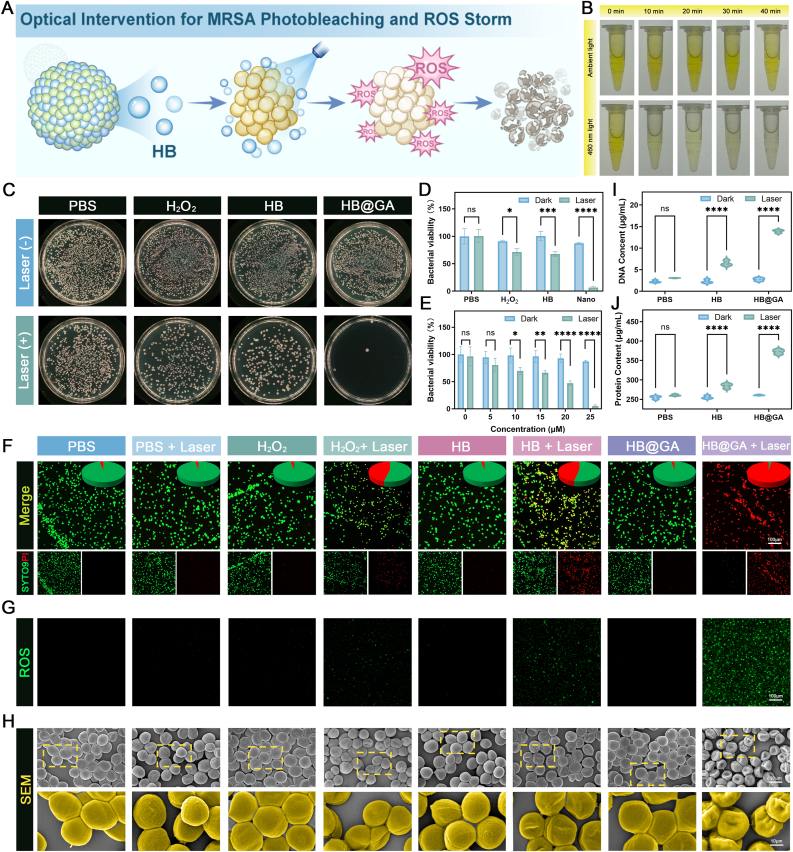
*In vitro* antibacterial effects of HB@GA. (A) Schematic illustration of the proposed mechanism: 460 nm laser-induced photobleaching of staphyloxanthin (STX) sensitizes MRSA to reactive oxygen species (ROS), thereby potentiating HB-mediated photodynamic killing. (B) Colorimetric assessment of STX photobleaching. Crude STX extracts were exposed to either 460 nm laser light or ambient light for 0 to 40 min, demonstrating time-dependent fading only under laser irradiation. (C) Representative photographs showing the bacterial colony counts under different treatment conditions on hypoxia conditions. (D) Bacterial viability of MRSA treated with PBS, H_2_O_2_, HB, or HB@GA under dark conditions and laser exposure. (E) Bacterial viability of MRSA treated with varying concentrations of HB@GA under dark conditions and laser exposure. (F) Representative images of the Syto 9/PI-stained MRSA after various treatments. (G) Representative images of DCF fluorescence in MRSA illustrating intracellular ROS levels following various treatments. (H) SEM images of MRSA upon various treatments. (I) Protein and (J) DNA content of MRSA supernatant after various treatments. Data are expressed as mean ± SD (n = 3). The statistical significance was calculated using a two-way ANOVA, ∗∗p < 0.01, ∗∗∗p < 0.001, ∗∗∗∗p < 0.0001.

To systematically evaluate the antibacterial performance of the HB@GA nanotherapeutic, logarithmic-phase methicillin-resistant *Staphylococcus aureus* (MRSA) was used as the model pathogen. The proposed therapeutic mechanism, illustrated in Fig. 4A, involves a synergistic two-step action where 460 nm laser light first photobleaches the bacterial antioxidant staphyloxanthin (STX) to sensitize the pathogen, thereby potentiating the subsequent photodynamic killing mediated by HB.

We first verified the photobleaching effect of 460 nm light on STX, the key antioxidant pigment of MRSA. As shown in Fig. 4B, a crude STX extract exhibited a homogeneous yellow color in the absence of 460 nm laser exposure, indicating the intact presence of the pigment. Upon illumination at 460 nm, the yellow color gradually faded with increasing irradiation time, eventually becoming colorless, which visually demonstrated the progressive degradation of STX. This direct visual observation, supported by quantitative absorption spectroscopy (Fig. S4), confirms the high photosensitivity of STX to 460 nm light and its efficient photobleaching. The removal of this bacterial antioxidant barrier establishes a critical precondition for enhancing the subsequent efficacy of HB-mediated photodynamic therapy.

To further validate the specific contribution of STX photobleaching at 460 nm to biofilm disruption, we performed comparative experiments using a control wavelength of 660 nm, which also matches an absorption peak of HB but does not efficiently photobleach STX. A biofilm eradication assay was conducted using crystal violet staining. As presented in Figs. S4 and S5, biofilms treated with PBS alone, PBS with 660 nm laser, PBS with 460 nm laser, or HB@GA alone remained largely intact with no significant reduction in biofilm mass. Treatment with HB@GA plus 660 nm laser resulted in partial biofilm disruption, presumably due to HB-mediated photodynamic activity alone. Strikingly, only the HB@GA plus 460 nm laser group led to near-complete biofilm destruction, as evidenced by the markedly decreased crystal violet staining (Fig. S5) and the lowest optical density values (Fig. S6). This superior efficacy was visually confirmed by live/dead fluorescent staining of biofilm-embedded bacteria (Fig. S7), where intense red fluorescence indicative of membrane-compromised dead cells was observed exclusively in the HB@GA plus 460 nm laser group. These results collectively demonstrate that the 460 nm light specifically enhances the antibiofilm effect by photobleaching STX, thereby sensitizing the pathogen to concurrent HB-mediated photodynamic assault.

The anti-MRSA efficacy of the HB@GA nanoplatform was systematically evaluated. Representative photographs of colony-forming units (CFU) revealed that near-complete bacterial eradication was achieved only when MRSA was treated with HB@GA followed by 460 nm laser irradiation (Fig. 4C). Quantitative viability assays further corroborated these findings (Fig. 4D). Neither free HB nor HB@GA exhibited significant antibacterial activity in the absence of light. Upon laser irradiation, both formulations demonstrated marked antibacterial effects, with HB@GA showing superior potency compared to free HB. This enhanced activity is likely attributable to the improved aqueous dispersion and cellular delivery of the hydrophobic HB photosensitizer facilitated by its co-assembly with GA. The antibacterial effect of HB@GA was found to be concentration-dependent, with a significant reduction in bacterial survival observed with increasing nanoparticle concentration (Fig. 4E). A concentration of 25 μM was identified as optimally effective and was therefore selected for subsequent investigations.

To further validate that the photobleaching of STX sensitizes MRSA to oxidative attack, we introduced hydrogen peroxide (H_2_O_2_) as a model ROS agent. The concentration of H_2_O_2_ was maintained at 100 μM to simulate physiological levels found in infected microenvironments. Notably, neither 460 nm light alone nor H_2_O_2_ alone exhibited antibacterial activity, whereas their combination resulted in a statistically significant reduction in bacterial viability (Fig. 4D). This result confirms that the photodegradation of STX by 460 nm light effectively compromises the intrinsic antioxidant defense of MRSA, thereby heightening its susceptibility to ROS. Direct visual evidence of bacterial viability was provided by a live/dead fluorescent staining assay as shown in Fig. 4F. Emerging red fluorescence indicative of compromised membranes and bacterial death was observed in the group treated with hydrogen peroxide and laser irradiation. Notably, the MRSA cells in the HB@GA plus laser group exhibited an almost ubiquitous and intense red fluorescence. This observation confirms the potent and light-activated bactericidal efficacy of the nanotherapeutic platform.

The mechanism underlying the observed bactericidal effect was further investigated. Intracellular reactive oxygen species (ROS) generation, a key mediator of photodynamic action, was directly quantified using the DCFH-DA fluorescent probe (Fig. 4G). A detectable ROS signal was produced in the H_2_O_2_ plus laser group. This signal was markedly stronger in the HB plus laser group and became most intense in the HB@GA plus laser treatment, indicating that the nanoplatform triggered the most robust and efficient ROS production upon light activation. scanning electron microscopy offered ultrastructural insights into the bactericidal process (Fig. 4H). MRSA treated with HB@GA in the dark maintained a smooth and intact coccal morphology characteristic of healthy cells. In stark contrast, bacteria subjected to HB@GA plus laser treatment displayed severe morphological devastation including membrane wrinkling, structural collapse, and extensive pore formation accompanied by the visible leakage of cytoplasmic contents. This physical disintegration serves as the definitive cellular signature of irreversible and ROS-induced cytolysis. The leakage of intracellular macromolecules including proteins and DNA was further quantified (Fig. 4I and J). Significantly higher levels of leakage were detected exclusively in the groups treated with HB and laser or HB@GA and laser. The latter group induced the most substantial efflux of intracellular contents. This data indicates that the laser-triggered ROS generation acts as the primary driver for membrane permeabilization.

In summary, these *in vitro* assays conclusively demonstrate that HB@GA exerts potent and light-activated bactericidal activity against MRSA. The mechanism involves a cascade of events initiated by superior ROS generation, enhanced by the GA-facilitated delivery and activation of HB. This oxidative burst overwhelms the bacterial antioxidant defenses, which are partially sensitized by the concurrent photobleaching of STX, leading to catastrophic membrane damage, leakage of cellular constituents, and ultimately, bacterial cell death.

### 3.4 *In vitro* immunomodulatory evaluation of HB@GA

**Fig. 5:**
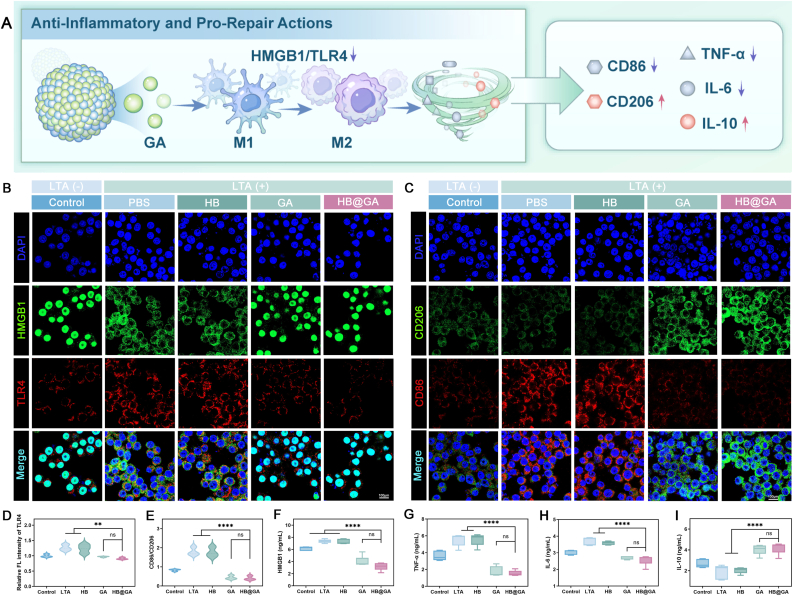
*In vitro* evaluation of the immunomodulatory effects of HB@GA on macrophages. (A) Schematic illustration of the anti-inflammatory effect of GA on RAW264.7 macrophages. (B) Representative immunofluorescence staining images showing the expression and subcellular localization of High Mobility Group Box 1 (HMGB1) and Toll-like Receptor 4 (TLR4) in RAW264.7 murine macrophages following various treatments. (C) Representative immunofluorescence staining images depicting the expression of the surface markers CD86 (M1 phenotype) and CD206 (M2 phenotype) in RAW264.7 macrophages, indicating their polarization state. (D) Quantitative analysis of TLR4 fluorescence intensity. (E) Quantitative analysis of the CD86/CD206 fluorescence ratio. (F-I) Quantitative analysis of pro-inflammatory and anti-inflammatory cytokines released into the culture supernatant by RAW264.7 cells, as determined by enzyme-linked immunosorbent assay (ELISA). The concentrations of (F) HMGB1, (G) Tumor Necrosis Factor-α (TNF-α), (H) Interleukin-6 (IL-6), and (I) Interleukin-10 (IL-10) are presented for different treatment groups. Data are expressed as mean ± SD (n = 3). The statistical significance was calculated using a one-way ANOVA, ∗∗p < 0.01, ∗∗∗p < 0.001, ∗∗∗∗p < 0.0001.

To simulate the *in vivo* ocular microenvironment and evaluate the immunomodulatory capacity of HB@GA across the corneal barrier, a transwell co-culture model was employed. Human corneal epithelial (HCE-T) cells were seeded in the upper chamber to mimic the corneal epithelium, while RAW264.7 murine macrophages were cultured in the lower chamber. Macrophages were first polarized to a pro-inflammatory M1 state using lipoteichoic acid (LTA, 5 μg/mL). This model allowed us to investigate whether HB@GA could traverse the epithelial barrier and modulate underlying macrophage function to promote a repair-favorable state. The proposed mechanism for this immunomodulation, centered on the anti-inflammatory action of GA, is schematically illustrated in Fig. 5A.

Before evaluating the immunomodulatory effects, we assessed the biocompatibility of HB@GA toward the corneal epithelial cells themselves. A CCK-8 assay was performed on HCE-T cells treated with increasing concentrations of HB@GA (Fig. S8). At the therapeutic concentration of 25 μM, the cell viability remained above 90%, indicating negligible cytotoxicity. Additionally, intracellular ROS levels in HCE-T cells were measured using the DCFH-DA probe (Fig. S9). The HB@GA alone group displayed ROS levels comparable to the untreated control, confirming that the nanocomplex does not induce oxidative stress in the absence of laser irradiation. In contrast, the positive control group treated with hydrogen peroxide showed a marked three to five fold increase in fluorescence. These results collectively confirm the excellent safety profile of HB@GA toward corneal epithelial cells, which is essential for its application as a topical ophthalmic formulation.

We next examined the effect of HB@GA on the HMGB1/TLR4 signaling axis, a central pathway through which the damage-associated molecular pattern (DAMP) HMGB1 initiates and sustains inflammatory responses. As shown in Fig. 5B, in control cells, HMGB1 was predominantly localized within the nucleus, with minimal TLR4 activation. Both the LTA and free HB groups exhibited marked cytoplasmic translocation of HMGB1 accompanied by strong TLR4 co-localization and activation, confirming the induction of a pro-inflammatory state. In contrast, treatment with either free GA or HB@GA effectively suppressed this LTA-driven response. Notably, the HB@GA nanotherapeutic demonstrated the most potent inhibitory effect, with HMGB1 largely retained in the nucleus and TLR4 signal substantially reduced, indicating a successful blockade of this key inflammatory pathway. Quantitative analysis of TLR4 fluorescence intensity (Fig. 5D) further corroborated these observations, revealing a significant reduction in the HB@GA group compared to LTA and free HB treated groups, confirming the superior blockade achieved by the nanotherapeutic.

Concurrently, phenotypic reprogramming of macrophages was directly visualized via laser scanning confocal microscopy for surface marker expression (Fig. 5C). The expression of CD86, a marker for the pro-inflammatory and bactericidal M1 phenotype, was significantly elevated in LTA and HB-treated groups, while CD206, a marker for the anti-inflammatory and tissue-reparative M2 phenotype, remained low. This pattern confirms that free HB alone does not possess inherent anti-inflammatory or tissue-repair functions. In contrast, treatments with GA or HB@GA effectively reversed this phenotype, demonstrating decreased CD86 and increased CD206 expression, which signifies a clear shift toward an M2-dominant state. These results highlight the critical role of GA in mediating the immunomodulatory switch and demonstrate that within the HB@GA nanoparticles, GA effectively drives macrophage reprogramming toward a pro-healing phenotype. Quantitative analysis of the CD86/CD206 ratio (Fig. 5E) confirmed that HB@GA treatment led to a significantly lower ratio compared to LTA and free HB groups, providing statistical evidence for the phenotypic shift toward the M2 reparative state.

The observed phenotypic shifts were further substantiated by quantitative analysis of secreted cytokines via ELISA (Fig. 5F–I). Groups treated with LTA or free HB exhibited significantly elevated levels of pro-inflammatory mediators, including HMGB1, TNF-α, and IL-6. In contrast, both free GA and the HB@GA nanotherapeutic markedly suppressed the release of these inflammatory signals. Notably, the HB@GA group uniquely and significantly enhanced the secretion of the anti-inflammatory cytokine IL-10. These results confirm that the nanocomplex not only potently inhibits key pro-inflammatory pathways but also actively fosters an immunoregulatory microenvironment that favors inflammation resolution.

In summary, the *in vitro* studies clearly establish the superior anti-inflammatory and immunomodulatory capacity of GA. The HB@GA nanoparticles function through a coordinated dual mechanism. First, it suppresses the initiation of the HMGB1/TLR4-driven inflammatory cascade. Second, it reprograms macrophage polarization from a pro-inflammatory M1 state toward a pro-repair M2 phenotype. This synergistic immunomodulatory action at the cellular level aligns with the design of a comprehensive “anti-infection and pro-repair” therapeutic strategy.

### 3.5 Mechanism of GA-mediated trans-corneal permeation

**Fig. 6:**
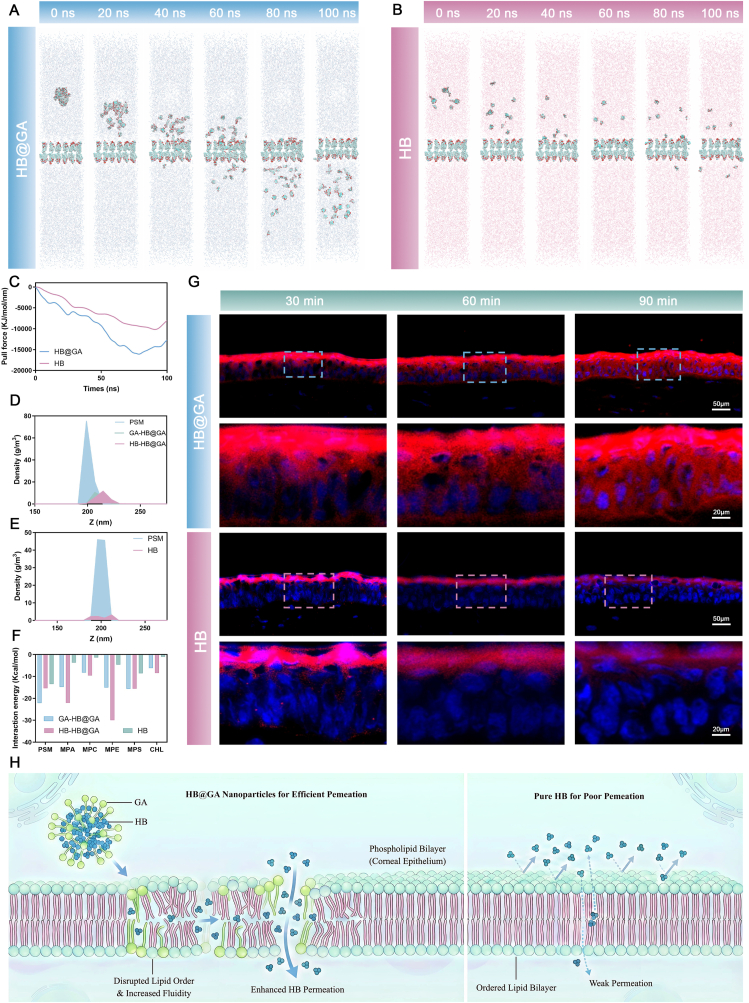
Computational and Experimental Analysis of HB@GA Nanoparticle Permeation through the Corneal Barrier. Dynamic snapshots illustrating the permeation of (A) HB@GA and (B) HB alone across a model corneal phospholipid membrane, with insets showing localized regions of membrane interaction. (C) Calculated pulling force profiles required to translocate HB@GA and HB across the membrane model. (D) Density distributions along the Z-axis for phospholipid membrane (PSM), HB, and GA components within the HB@GA nanotherapeutic. (E) Comparative density distributions of PSM and HB along the Z-axis in the HB-alone system. (F) Interaction energies between the phospholipid membrane and key components: GA-HB@GA, HB-HB@GA, and HB alone, derived from the two simulation models. (G) Representative fluorescence images depicting the penetration depth of HB@GA and HB within ex vivo mouse corneal tissue. (H) Schematic representation of the proposed “HB@GA-enhanced phospholipid membrane disruption toolkit” mechanism.

Overcoming the formidable barrier of the intact corneal epithelium is a prerequisite for effective ocular drug delivery [[42], [43], [44]]. To elucidate the molecular rationale behind the superior penetrability of HB@GA NPs, we conducted a multiscale investigation, bridging atomistic simulations with macroscopic tissue distribution. All-atom molecular dynamics (MD) simulations on a model corneal epithelial bilayer unveiled a striking dichotomy in translocation behavior (Fig. 6A and B). While free HB molecules were predominantly entrapped at the membrane-water interface, failing to breach the hydrophobic core, the HB@GA supramolecular assembly demonstrated a rapid, thermodynamically favorable translocation across the bilayer within 100 ns.

Quantitative analysis of the translocation free energy landscape via the potential of mean force (PMF) provided direct thermodynamic evidence for the enhancement mechanism. The PMF profile revealed a substantially lowered energy barrier for HB translocation within the HB@GA complex compared to free HB (Fig. 6C), indicating that GA co-assembly effectively reduces the thermodynamic penalty for transmembrane passage. To decipher the structural origin of this facilitation, density distribution analyses along the membrane normal were performed (Fig. 6D and E). GA exhibited preferential accumulation at the lipid headgroup region (phosphatidylserine monolayer, PSM), while concurrently enabling a pronounced increase in HB density within the hydrophobic core of the bilayer. This distinct spatial partitioning suggests GA functions as an interfacial modulator. Further quantitative interaction energy calculations confirmed that GA significantly strengthens the binding affinity between HB and key hydrophobic lipid regions (myristic acid, MPA; myristic acid phosphatidylethanolamine, MPE) (Fig. 6F). Collectively, these simulations delineate a coherent mechanism wherein GA, by interfering the lipid-water interface, remodels local membrane thermodynamics and enhances specific drug-lipid interactions, thereby catalyzing the penetration of its co-assembled HB payload.

These molecular-scale predictions were rigorously validated *in vivo*. Following topical administration in a murine model, spatiotemporal tracking of HB distribution revealed a profound difference (Fig. 6G). The HB@GA nanotherapeutic displayed sustained, time-dependent penetration deep into the corneal stroma, with fluorescence intensity progressively increasing over 90 min. In stark contrast, free HB was largely confined to the superficial epithelium, with its signal rapidly diminishing due to pre-corneal clearance. This *in vivo* outcome directly corroborates the simulations, confirming that the GA-mediated reduction of the interfacial energy barrier translates into significantly enhanced and prolonged corneal delivery.

Integrating computational and experimental evidence, we propose a novel mechanistic paradigm, schematized in Fig. 6H. Within the supramolecular nanocomplex, GA acts as a multifunctional permeation enhancer. Its interfacial localization serves to modulate the thermodynamics of the membrane-solution interface, while its amphiphilic structure synergizes with HB to induce transient, mild perturbation of the hydrophobic lipid packing. This dual-scale mechanism, combining interfacial catalysis with subtle membrane restructuring, represents a sophisticated and efficient strategy for modulating phospholipid bilayer permeability. Unlike the passive diffusion of free drug, this actively engineered, biomimetic mechanism equips the HB@GA nanotherapeutic with the unique capability to effectively overcome the corneal epithelial barrier. This breakthrough not only ensures high topical bioavailability but also establishes a promising platform for effective non-invasive therapy of posterior segment diseases.

### 3.6 *In vivo* eradication of MRSA keratitis via HB@GA-potentiated PDT

**Fig. 7:**
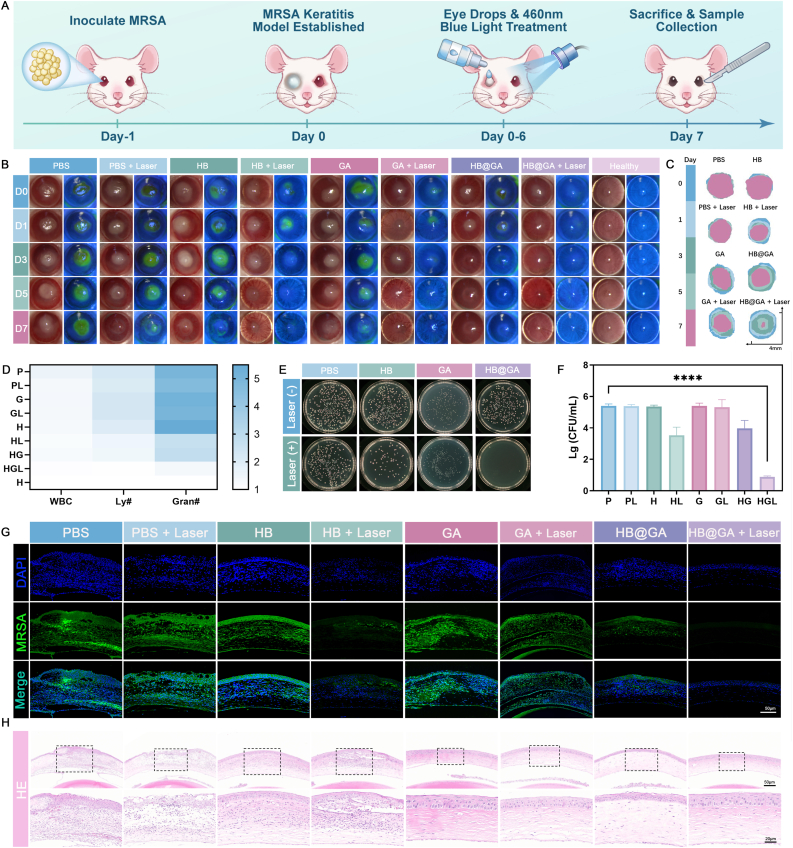
Antibacterial effects of HB@GA-based PDT *in vivo*. (A) Schematic illustration of *in vivo* antibacterial experiment. (B) Slit-lamp examination images of mouse corneas under white light and Fluorescein sodium staining images of mouse corneas under cobalt blue light slit-lamp examination. (C) Schematic diagram illustrating changes in corneal bacterial plaque size. (D) Heatmap generated from complete blood count (CBC) analysis. (E) Representative photographs of agar plates showing bacterial colony counts from homogenized eyeballs across different treatment groups on Day 7. (F) Quantitative analysis of bacterial counts in corneal tissue homogenates. (G) Fluorescence images of corneal sections stained with DAPI (4′,6-diamidino-2-phenylindole; blue) and an MRSA-specific probe (FITC; green). (H) Hematoxylin and eosin (H&E) stained images of mouse corneal tissues. Data are expressed as mean ± SD (n = 5). The statistical significance was calculated using a one-way ANOVA, ∗∗p < 0.01, ∗∗∗p < 0.001, ∗∗∗∗p < 0.0001. (For interpretation of the references to color in this figure legend, the reader is referred to the Web version of this article.)

Encouraged by the robust *in vitro* bactericidal performance, we evaluated the therapeutic potential of the HB@GA nanotherapeutic in a murine model of MRSA-induced bacterial keratitis (Fig. 7A). Clinical progression, monitored via serial slit-lamp examinations, revealed distinct therapeutic outcomes (Fig. 7B). Control groups (PBS and HB without laser) succumbed to severe infection, manifesting as dense corneal opacity, extensive ulceration, and purulent discharge. Fluorescein sodium staining, which highlights epithelial defects by binding to exposed stroma and emitting yellow fluorescence under cobalt blue light, confirmed extensive corneal breakdown in these groups.

Interestingly, the PBS + Laser group exhibited a modest alleviation of pathology. We attribute this effect to a specific photo-mechanism, wherein 460 nm laser irradiation likely induces photobleaching of STX, the characteristic antioxidant pigment of MRSA. This process is postulated to sensitize the bacteria to oxidative stress within the inflammatory microenvironment. The HB@GA (dark) group also demonstrated measurable symptom mitigation, an outcome consistent with the known immunomodulatory and reparative properties of GA. Notably, both the GA alone and GA + Laser groups showed partial clinical improvement, characterized by reduced corneal opacity and some resolution of epithelial defects. This recovery is attributed to the intrinsic anti-inflammatory and tissue-repair functions of GA, which promote healing independent of bacterial clearance. However, the most striking and complete recovery was observed exclusively in the HB@GA + Laser group, where corneas were rapidly restored to a transparent, healthy state with negligible fluorescein retention. This superior efficacy is driven by a coordinated “access-and-attack” strategy. Here, GA facilitates deep stromal accumulation of the photosensitizer, as established in Section 3.4, while concurrently exerting intrinsic anti-inflammatory effects. Together, these actions establish a tissue environment conducive to the efficient photodynamic eradication of deep-seated MRSA by activated HB.

Systemic and local analyses on Day 7 corroborated the clinical observations. Hematological profiling indicated that only the HB@GA + Laser group displayed normalized white blood cell (WBC), lymphocyte (Lymph), and granulocyte (Gran) counts, which were indistinguishable from those of uninfected healthy controls, signaling the resolution of systemic inflammation (Fig. 7D). The GA and GA + Laser groups showed partial reduction in leukocyte counts compared to PBS controls, reflecting the anti-inflammatory effect of GA, but their values remained above normal due to persistent infection. All other groups exhibited persistent leukocytosis. Quantitative bacteriological assessment of ocular homogenates provided definitive evidence of bacterial clearance (Fig. 7E and F). While PBS + Laser and HB + Laser treatments led to minor and moderate reductions in colony-forming units (CFUs), respectively, only the HB@GA + Laser treatment achieved complete bacterial eradication. Notably, the GA and GA + Laser groups showed no significant reduction in bacterial load compared to the PBS control, confirming that GA alone or combined with laser does not possess direct antibacterial activity. This result unequivocally underscores the indispensable role of GA in enabling sufficient corneal penetration and subsequent activation of the HB photosensitizer to attain a microbiological cure, while its independent effect is limited to immune modulation and tissue repair.

The absence of viable bacteria was visually confirmed by dual-fluorescence staining of corneal sections with DAPI and an MRSA-specific FITC probe, which showed negligible signal in the HB@GA + Laser group in stark contrast to the high bacterial loads observed in all other groups, including GA and GA + Laser (Fig. 7G). In the GA and GA + Laser groups, green fluorescence indicative of MRSA remained abundant, further demonstrating that GA lacks intrinsic bactericidal activity despite its reparative effects. Finally, histopathological evaluation via hematoxylin and eosin (H&E) staining provided structural evidence of tissue recovery (Fig. 7H). Corneas from control and monotherapy groups displayed severe edema, epithelial defects, and dense inflammatory infiltrates. The GA and GA + Laser groups exhibited moderate improvement, with reduced edema and inflammatory cell infiltration but residual structural abnormalities, reflecting GA-mediated tissue repair in the absence of infection control. In marked contrast, corneas treated with HB@GA + Laser were largely restored to a normal histological architecture, featuring an intact epithelium, resolved stromal edema, and a notable absence of inflammatory cells. Collectively, these data establish HB@GA-based PDT as a sophisticated, antibiotic-free modality that synergizes deep-tissue permeation, targeted phototoxicity, and immunomodulation to effectively manage refractory ocular infections.

### 3.7 Immune microenvironment reprogramming via HB@GA-mediated PDT

**Fig. 8:**
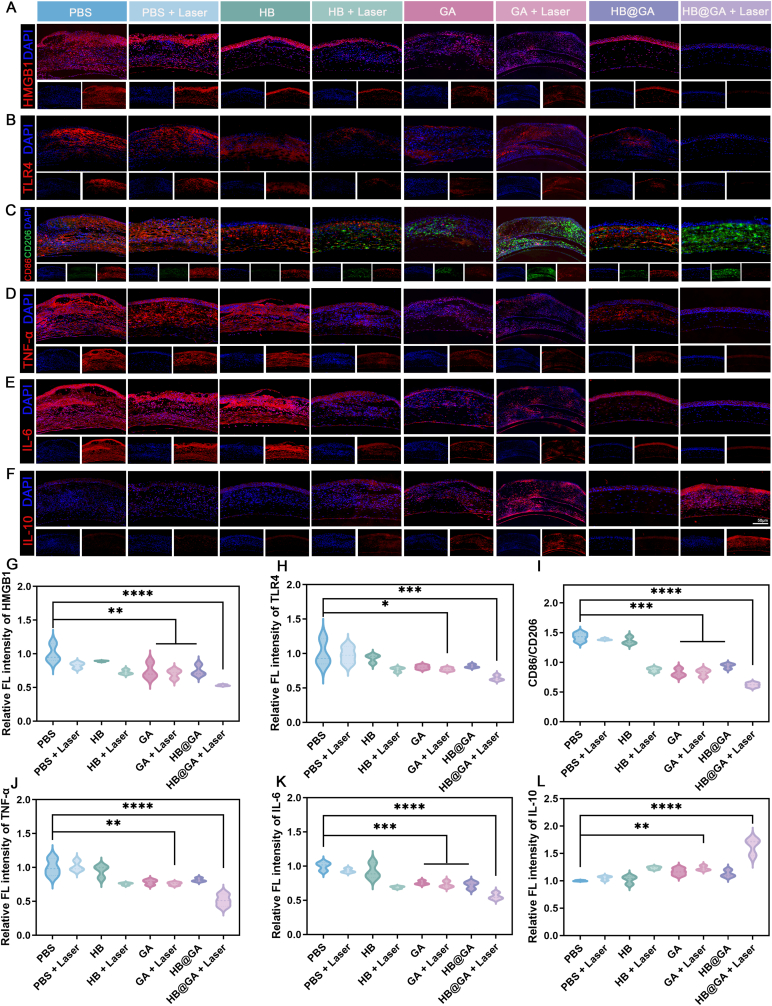
*In vivo* anti-inflammatory action of HB@GA-based photodynamic therapy (PDT) in MRSA-induced bacterial keratitis. (A-F) Representative immunofluorescence staining images depicting the expression of key inflammatory mediators in corneal tissues from different treatment groups on day 7 post-infection: (A) HMGB1, (B) TLR4, (C) the macrophage polarization markers CD86 and CD206, (D) TNF-α, (E) IL-6, and (F) IL-10. Images were acquired using consistent microscopy parameters across all samples. (G-K) Corresponding quantitative statistical analysis of the fluorescence intensity for (G) HMGB1, (H) TLR4, (I) the CD86/CD206 expression ratio, (J) TNF-α, (K) IL-6 and (L) IL-10, as determined by quantitative image analysis of the stained tissue sections. Data are expressed as mean ± SD (n = 3). The statistical significance was calculated using a one-way ANOVA, ∗∗p < 0.01, ∗∗∗p < 0.001, ∗∗∗∗p < 0.0001.

Beyond bactericidal efficacy, the restoration of corneal transparency necessitates the active resolution of infection-induced inflammation. We therefore profiled the immunomodulatory landscape on day 7 to decipher how HB@GA-based PDT reprograms the host immune response (Fig. 8A–F). Quantitative analysis revealed that the treatment does not merely suppress inflammation but actively steers the immune trajectory from a destructive catabolic state toward a pro-regenerative anabolic phase.

Mechanistically, the therapeutic intervention strategically targets the apex of the inflammatory cascade by disrupting the critical HMGB1-TLR4 signaling axis. In the context of bacterial keratitis, the release of HMGB1, a potent damage-associated molecular pattern (DAMP) that amplifies and perpetuates inflammatory responses, serves as a key driver of persistent tissue damage and impaired healing. In the HB@GA + Laser group, HMGB1 expression was significantly suppressed (Fig. 8A–G). This reduction was accompanied by a synchronized downregulation of its cognate receptor TLR4 (Fig. 8B–H). Notably, the GA alone and GA + Laser groups also exhibited modest reductions in HMGB1 and TLR4 levels compared to the PBS control, reflecting the intrinsic anti-inflammatory capacity of GA. However, these reductions were substantially less pronounced than those observed in the HB@GA + Laser group, indicating that the combination with HB and laser is required for full pathway blockade. By dismantling this upstream “danger signal” circuit, HB@GA-PDT effectively intercepts the propagation of the inflammatory cascade, thereby mitigating downstream tissue damage and creating a microenvironment conducive to structured repair.

The strategic disruption of the HMGB1-TLR4 axis, a master regulator of innate immune activation, precipitated a decisive reprogramming of the local immune landscape. By intercepting this primary danger signaling cascade, the therapy fundamentally redirected macrophage polarization, a pivotal checkpoint governing the transition from inflammatory escalation to active resolution. Immunofluorescence analysis corroborated this host-directed immunomodulation, revealing a significant decrease in the CD86/CD206 ratio within the HB@GA + Laser group (Fig. 8C and I). This shift in biomarker ratio underscores a concerted phenotypic transition from a pro-inflammatory, bactericidal M1 profile (marked by CD86) toward an anti-inflammatory, tissue-reparative M2 state (marked by CD206). The GA and GA + Laser groups showed a mild but detectable decrease in the CD86/CD206 ratio relative to the PBS control, consistent with their partial anti-inflammatory activity. Nonetheless, the magnitude of macrophage repolarization remained far lower than that achieved by the HB@GA + Laser regimen, reinforcing the need for simultaneous bacterial clearance to fully shift the immune setpoint. Such repolarization is instrumental in reshaping the wound microenvironment, effectively converting it from a state of host-damaging immune confrontation to one that is permissive and actively supportive of structured tissue regeneration.

The functional translation of this phenotypic switch was evident in the cytokine profile. The established M2-dominant landscape correlated with a significant suppression of classic pro-inflammatory effector cytokines. Levels of tumor necrosis factor-alpha (TNF-α) and interleukin-6 (IL-6) were markedly reduced in the HB@GA + Laser group compared to all control groups (Fig. 8D, E, J, K). The GA and GA + Laser groups exhibited intermediate reductions in these pro-inflammatory cytokines, which aligned with their weaker but still measurable anti-inflammatory effects. Concurrently, we observed a substantial upregulation of interleukin-10 (IL-10) (Fig. 8F and L). The elevation of this potent anti-inflammatory and pro-resolving cytokine, strongly associated with M2 macrophage activity, confirms the establishment of an active resolution program. In the GA groups, IL-10 levels were modestly elevated compared to controls but remained significantly lower than those in the HB@GA + Laser group, indicating that GA alone can partially tilt the balance toward resolution, yet maximal effect requires the full integrated therapy.

Collectively, these findings demonstrate that the therapeutic action of HB@GA + Laser extends beyond direct microbicidal activity to encompass a sophisticated form of host-directed immunomodulation. By disrupting the initial HMGB1-TLR4 danger signal, the treatment prevents the escalation of destructive inflammation. This upstream intervention facilitates the repolarization of macrophages toward an M2 phenotype, which in turn remodels the cytokine environment—suppressing pro-inflammatory mediators such as TNF-α and IL-6 while amplifying pro-resolution signals like IL-10. The inclusion of GA and GA + Laser groups reveals that GA contributes a baseline anti-inflammatory effect, but the full immunomodulatory and bactericidal synergy is only realized when GA is combined with HB and laser activation. This coordinated immunomodulation, achieved concurrently with complete pathogen eradication, effectively controls infection-associated immunopathology and creates a conducive microenvironment for corneal regeneration. This multi-faceted mechanism underpins the superior clinical and histological recovery observed with the HB@GA-based PDT platform.

### 3.8 Transcriptome analysis of cornea tissue

**Fig. 9:**
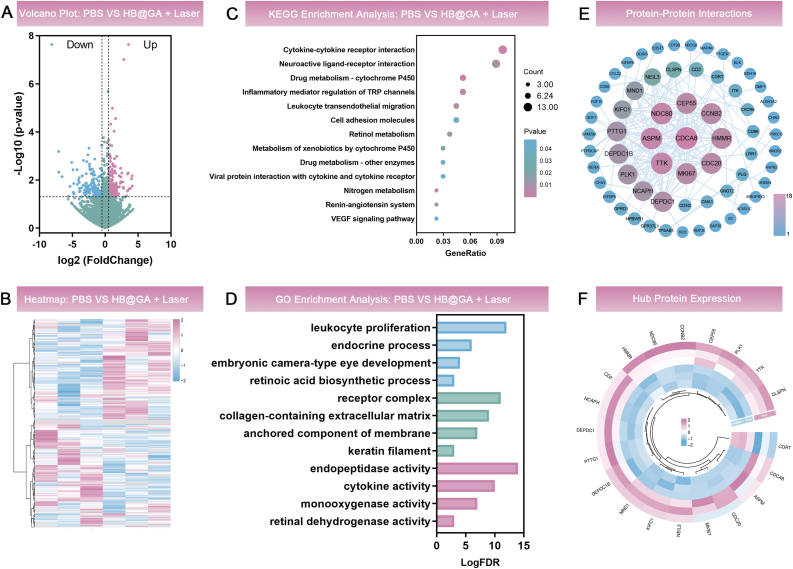
RNA-seq of cornea tissue treated with PBS and HB@GA + Laser. (A) Volcano map for the distribution of DEGs (green: genes that are not significantly changed; blue: down-regulated genes; and purple: up-regulated genes). (B) Heatmap of DEGs. (C) KEGG pathway analysis of DEGs. (D) GO analysis of DEGs. (E) Protein protein interactions of DEGs. (F) Heatmaps of Hub protein expression. (For interpretation of the references to color in this figure legend, the reader is referred to the Web version of this article.)

To decipher the molecular underpinnings driving the therapeutic efficacy of HB@GA-based photodynamic therapy (PDT), we performed comprehensive RNA sequencing (RNA-seq) on corneal tissues from mice treated with either PBS or HB@GA + Laser. This transcriptomic profiling revealed a profound reconfiguration of the host genetic landscape following treatment. A total of 314 differentially expressed genes (DEGs) were identified (|log2(fold change)| > 1, adjusted p-value <0.05)), with a balanced distribution of 161 downregulated and 153 upregulated genes (Fig. 9A). Unsupervised hierarchical clustering demonstrated a distinct segregation between the treatment and control groups, confirming that HB@GA + Laser induces a consistent and robust shift in global gene expression patterns distinct from the pathological state (Fig. 9B).

Kyoto Encyclopedia of Genes and Genomes (KEGG) pathway enrichment analysis unveiled a dual-modal therapeutic mechanism: immunomodulation and metabolic restoration (Fig. 9C). First, the therapy potently attenuated pathological inflammation. Enriched pathways, such as Cytokine-cytokine receptor interaction, Inflammatory mediator regulation of TRP channels, and Leukocyte transendothelial migration, suggest that HB@GA + Laser actively suppresses the cytokine storm and blocks excessive leukocyte infiltration, which are primary drivers of corneal haze and scarring. Second, the analysis highlighted a restoration of corneal homeostasis. The upregulation of metabolic pathways, including Retinol metabolism and Nitrogen metabolism, indicates a reactivation of essential physiological processes suppressed during infection. Notably, the enrichment of Drug metabolism-cytochrome P450 implies an enhanced capacity for detoxification and oxidative stress management in the healing tissue. Gene Ontology (GO) analysis further corroborated this pro-regenerative transition (Fig. 9D). In terms of molecular function and biological processes, terms such as retinal dehydrogenase activity and retinoic acid biosynthetic process were significantly enriched. Given the critical role of retinoic acid in corneal epithelial maintenance, this points to a targeted molecular program fostering epithelial integrity. Furthermore, cellular component terms like collagen-containing extracellular matrix and keratin filament underscore the structural remodeling of the stroma, critical for preventing corneal perforation and restoring transparency.

To identify core regulatory nodes within this transcriptional network, a protein-protein interaction (PPI) network was constructed from the DEGs. Topological analysis pinpointed 20 hub genes central to network connectivity, including CDCA8, NDC80, PLK1, and CCNB2 (Fig. 9E). A focused heatmap illustrating the expression levels of these hub genes vividly confirmed their differential regulation between treatment groups (Fig. 9F). The collective downregulation of multiple hub genes involved in cell cycle progression (e.g., CDCA8, ASPM, CCNB2) aligns with the anti-proliferative, pro-repair microenvironment fostered by HB@GA-PDT, diverting cellular energy from proliferation toward structured regeneration.

In summary, the transcriptomic landscape elucidates that HB@GA + Laser does not merely act as a bactericide but functions as an immunometabolic modulator. By simultaneously dampening nociceptive and inflammatory signaling while reinvigorating retinol metabolism and structural matrix synthesis, the therapy orchestrates a holistic healing environment, offering a superior strategy for managing severe bacterial keratitis.

### 3.9 *In vivo* biocompatibility and biosafety profile

Beyond therapeutic efficacy, the comprehensive assessment of biocompatibility is a prerequisite for the clinical translation of any nanomaterial, particularly for ocular administration where tissue sensitivity is high. We conducted a systematic safety evaluation of the HB@GA formulation in the murine model, including a Laser only group to specifically assess any potential phototoxicity of 460 nm irradiation on healthy ocular tissues.

Serum biochemical analysis demonstrated that key indicators of hepatic function (ALT, AST) and renal function (BUN, CRE) in the HB@GA treated group remained within physiological ranges, showing no statistically significant deviations compared to the PBS control group (Fig. S10). Importantly, the Laser only group also exhibited normal serum parameters, indicating no systemic toxicity from light exposure alone. Furthermore, major organs including the heart, liver, spleen, lungs, and kidneys were harvested for histopathological examination. H&E staining revealed well-preserved tissue architecture with no discernible signs of necrosis, inflammatory infiltration, or cellular lesions across all groups, including the Laser only and HB@GA treated groups, indistinguishable from the PBS control counterparts (Fig. S11).

To further evaluate potential oxidative stress and inflammatory responses induced by the treatments, we quantified serum levels of oxidative stress markers including malondialdehyde (MDA), superoxide dismutase (SOD), reduced glutathione (GSH), and catalase (CAT) (Fig. S11). No significant differences were observed among the PBS control, Laser only, and HB@GA treated groups, indicating that neither laser irradiation alone nor the HB@GA formulation provoked systemic oxidative imbalance. Similarly, serum levels of inflammatory cytokines such as HMGB1, TNF-α, IL-6, and IL-10 remained unchanged across all groups (Fig. S12), confirming the absence of systemic inflammatory activation.

Crucially, for ocular application, the corneal and surrounding tissues maintained structural integrity post-treatment, confirming that the therapeutic dosage of HB@GA plus Laser does not elicit collateral phototoxicity or irritation to healthy ocular structures. Collectively, these findings validate the excellent biosafety profile of the self-assembled HB@GA nanoplatform. The absence of acute systemic and local toxicity, attributed to the inherent biocompatibility of its natural building blocks, positions HB@GA as a promising, safe-by-design candidate for treating extensive ocular infections.

## 4 Conclusion

In summary, this study conceptually validates a “shattering the golden armor” strategy enabled by a fully bioactive supramolecular nanoplatform (HB@GA) for the precision management of MDR keratitis. Distinct from conventional antibiotic or PDT approaches, our design establishes a “one-photon, dual-action” paradigm that leverages 460 nm light as a molecular switch to simultaneously dismantle the bacterial antioxidant STX defense and unleash a lethal oxidative storm. The innovation of this work is highlighted by three promising features: (i) Minimalist yet Maximalist Design. By adopting a carrier-free supramolecular strategy, we eliminated inert excipients to achieve a 100% therapeutic payload, proving that natural building blocks can be engineered into sophisticated nanomedicines without synthetic complexity. (ii) Overcoming Dual Barriers. The amphiphilic nature of GA effectively unlocks the dense corneal epithelial barrier, while the optical disarming mechanism breaches the bacterial biochemical defense, addressing the fundamental recalcitrance of MRSA infections. (iii) From Kill-Only to Eradicate-and-Repair. Beyond sterilization, the HB@GA platform actively orchestrates corneal reconstruction. By modulating the HMGB1/TLR4 axis to drive macrophage reprogramming (M1-to-M2 transition), it resolves the therapeutic dilemma of post-infectious tissue damage. Collectively, the HB@GA nanoplatform represents a safe-by-design, antibiotic-free solution that integrates optical virulence modulation with immunometabolic repair. It provides a groundbreaking blueprint for addressing the complex dual challenges of biochemical resilience and physical barriers in multidrug-resistant infections, with broad implications for next-generation antimicrobial therapeutics.

## CRediT authorship contribution statement

**Zhi-heng Yang:** Conceptualization, Project administration, Supervision, Validation. **Hong-miao Dang:** Formal analysis, Writing – original draft. **Xiao Zhang:** Investigation, Methodology. **Ling-feng Xu:** Data curation. **Lu-lu Wang:** Data curation. **Xin Pang:** Conceptualization, Funding acquisition, Resources, Writing – review & editing. **You-hong Hu:** Supervision, Validation.

## Declaration of competing interest

The authors declare that they have no known competing financial interests or personal relationships that could have appeared to influence the work reported in this paper.

## Data Availability

Data will be made available on request.
