# Supplementary material for: Shattering the “golden armor”: A natural supramolecular nanoplatform for optical virulence modulation and MRSA keratitis therapy
Source: Mater Today Bio. 2026 Jun 22;39:103374. doi: 10.1016/j.mtbio.2026.103374 (PMC13320280; doi:10.1016/j.mtbio.2026.103374)
Supplement: Multimedia component 1 [file mmc1.docx]

**Supplementary Information**

**Shattering the “Golden Armor”: A Natural Supramolecular Nanoplatform for Optical Virulence Modulation and MRSA Keratitis Therapy**

*Zhi-heng Yang,^1^ Hong-miao Dang,^1^ Xiao Zhang, Ling-feng Xu, Lu-lu Wang, Xin Pang*, You-hong Hu**

Z.-H. Yang, H.-M. Dang, L.-L. Wang and Prof. Y.-H. Hu

Department of Pharmacy, The First Affiliated Hospital of Zhengzhou University, Zhengzhou, Henan 450052, China

L.-F. Xu and Prof. X. Pang

School of Pharmacy, Henan University of Chinese Medicine, Zhengzhou 450046, China.

X. Zhang

College of Chemical Engineering, Fuzhou University, Fujian 350108, China

^1^The authors contributed equally to this work.

*Correspondence authors

E-mail: pangxin116@163.com (X. Pang), hyouhong@163.com (Y.-H. Hu)


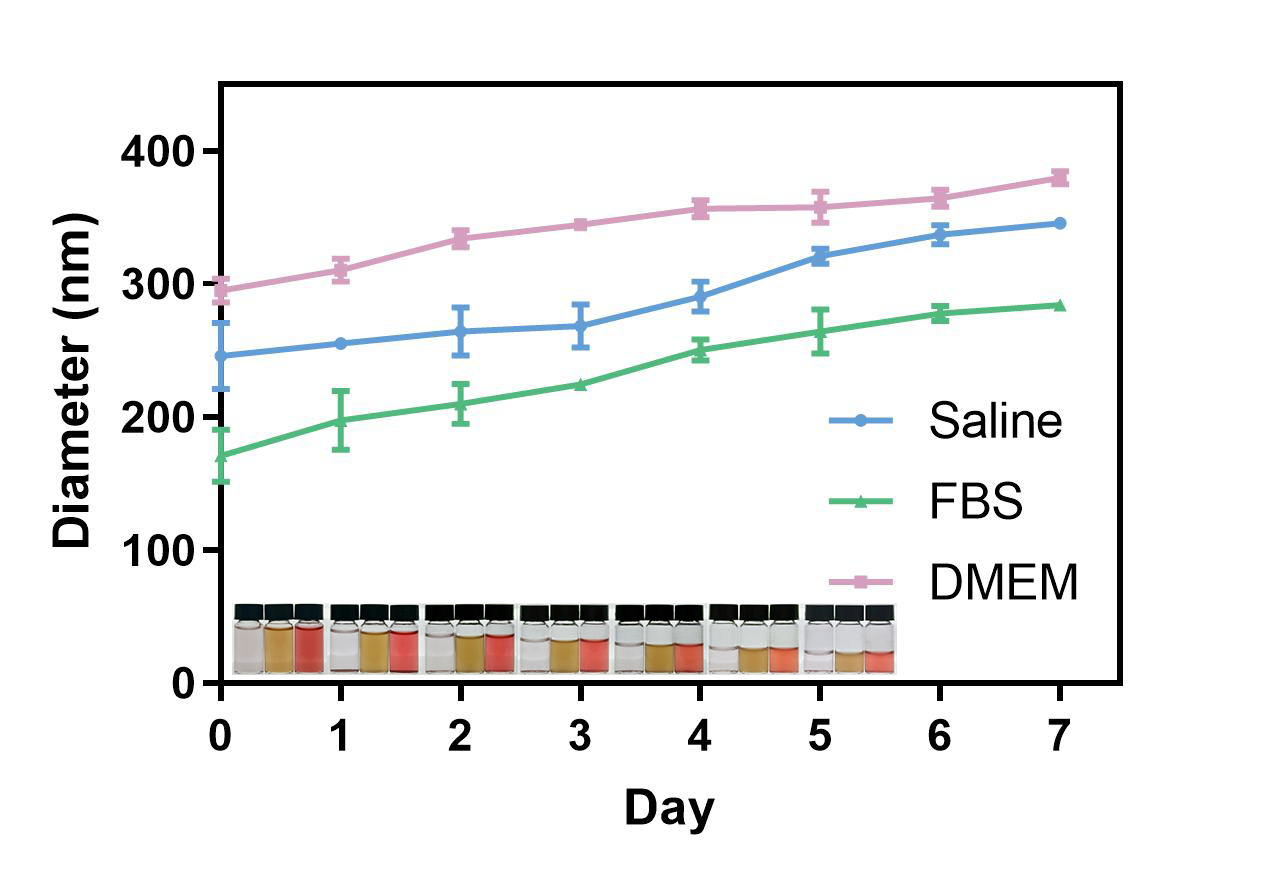


**Figure S1** Stability investigation of HB@GA nanoparticles in PBS, FBS, and DMEM over a 7-day period.


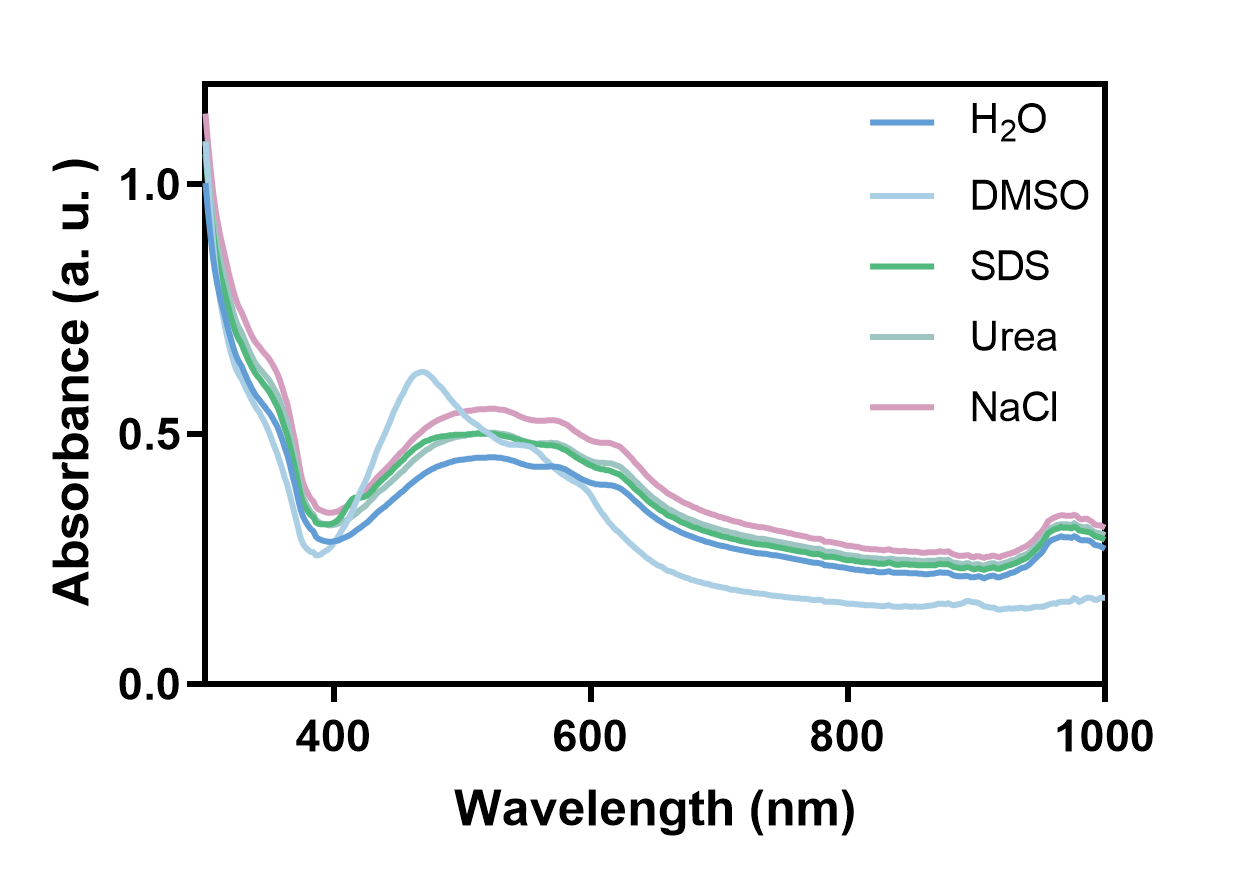


**Figure S2** UV-vis-NIR absorption spectra of nanoparticles in different media (water, urea, THF, SDS, NaCl).


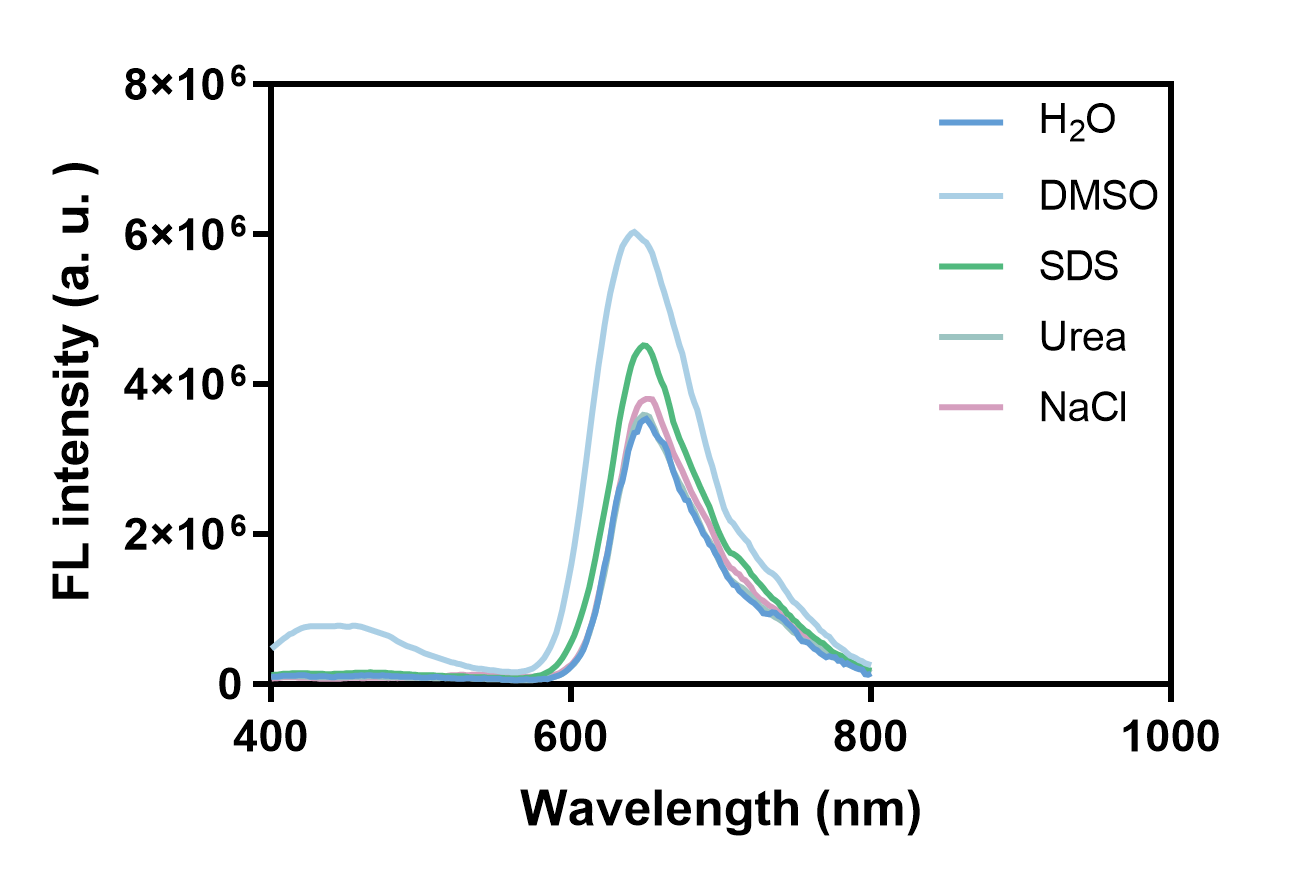


**Figure S3** Fluorescence spectra of nanoparticles in different media (water, urea, THF, SDS, NaCl).


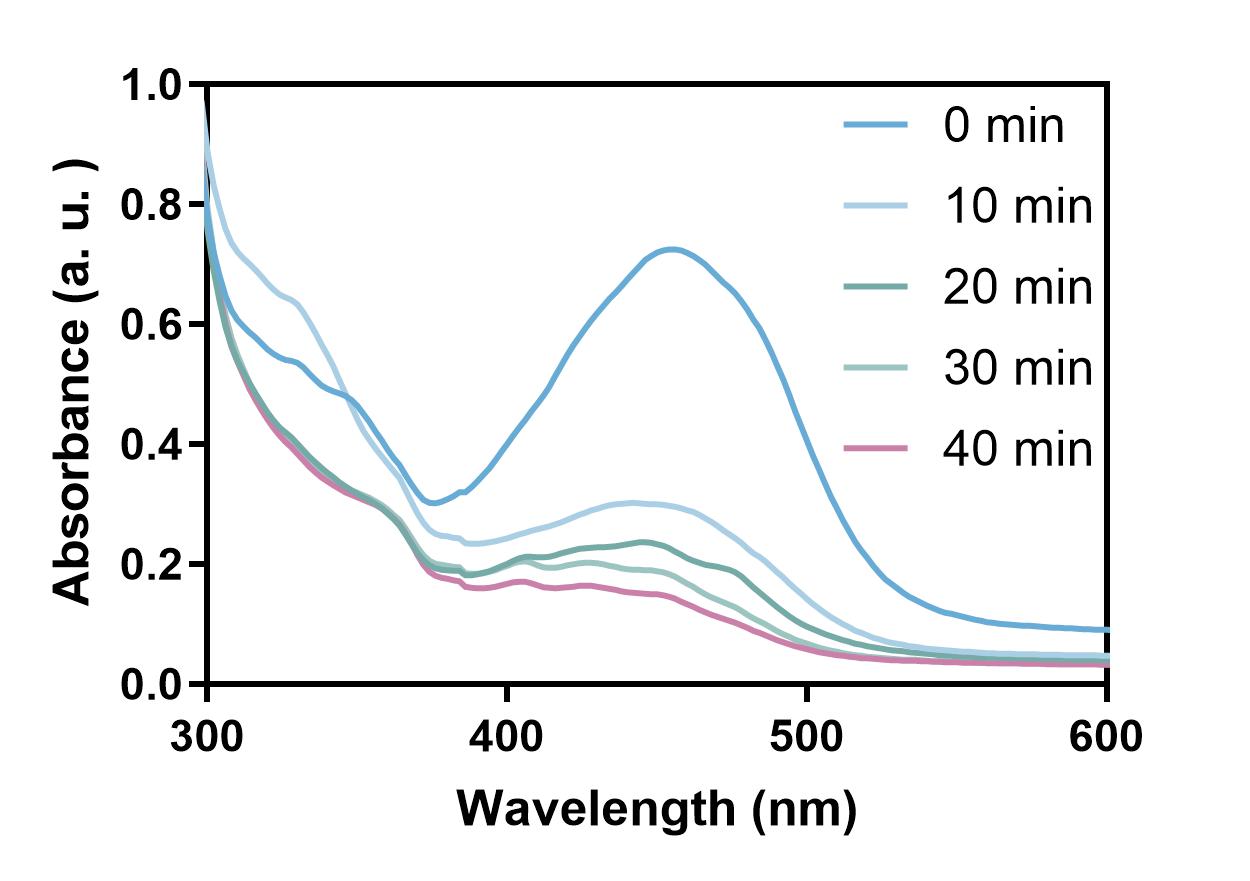


**Figure S4** Quantitative absorption spectra of STX following irradiation with 460 nm light.


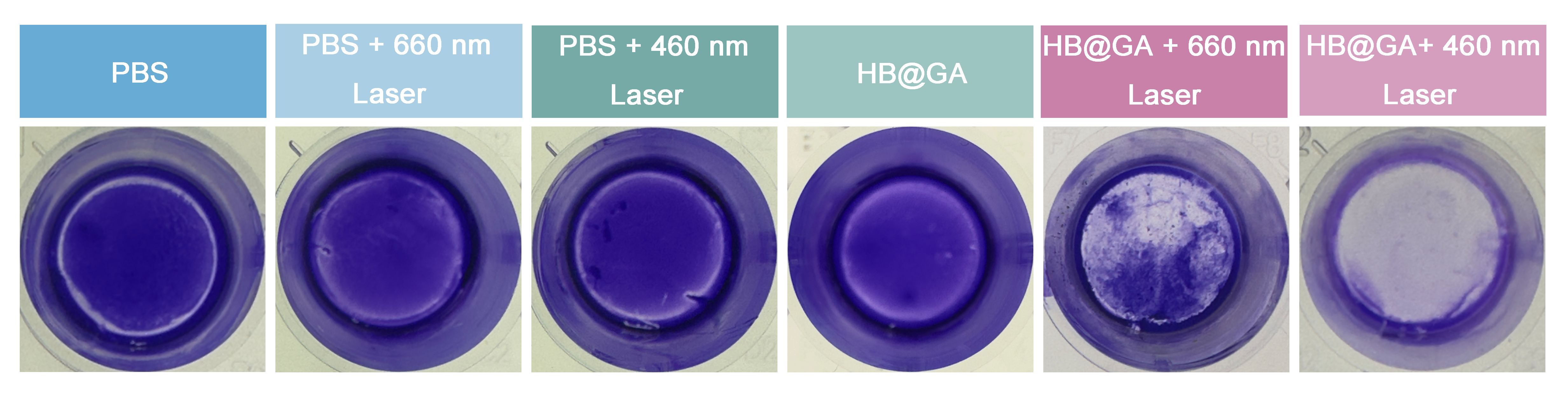


**Figure S5** Crystal violet staining photographs of bacterial biofilms treated with different groups.


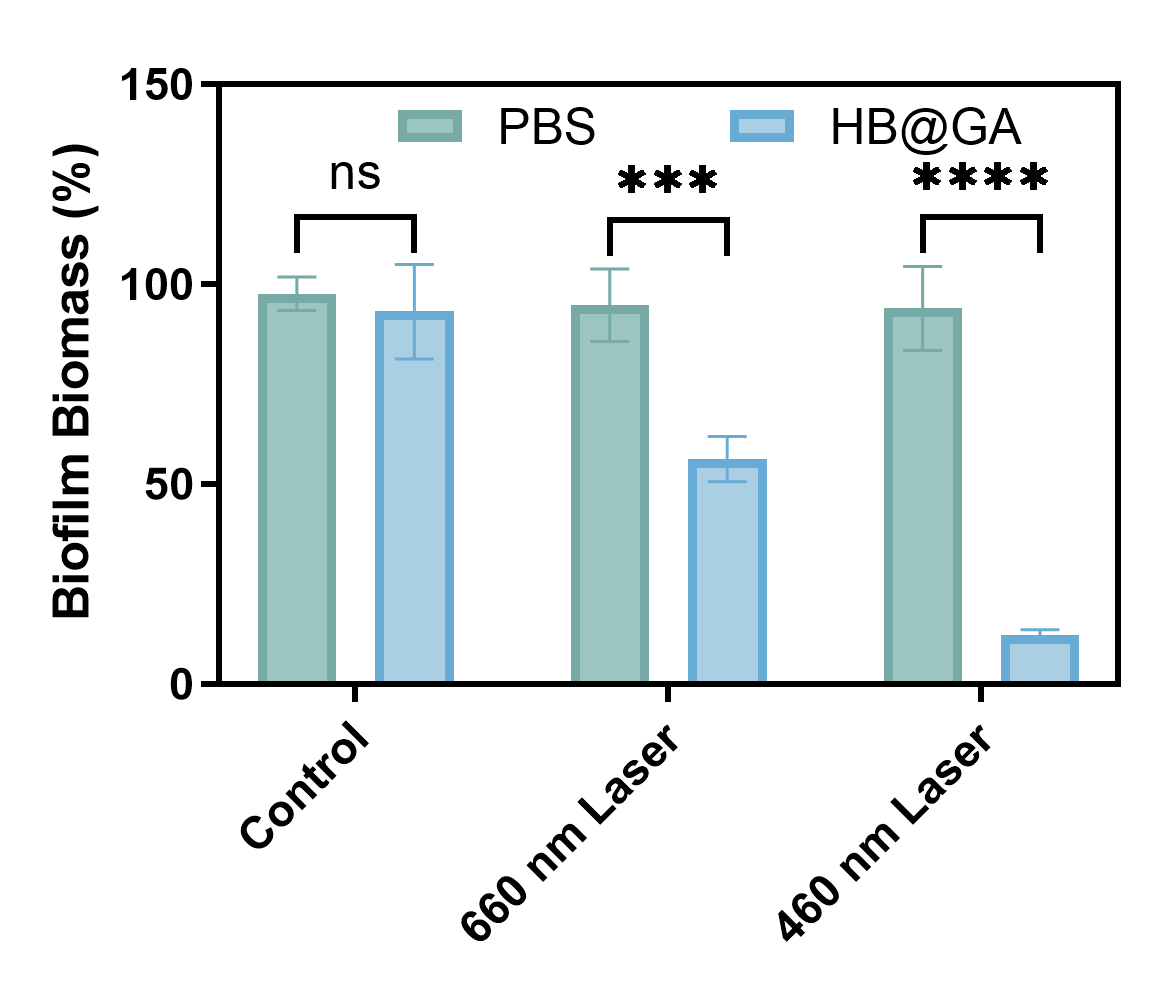


**Figure S6** Quantitative analysis of biofilm mass after different treatments.


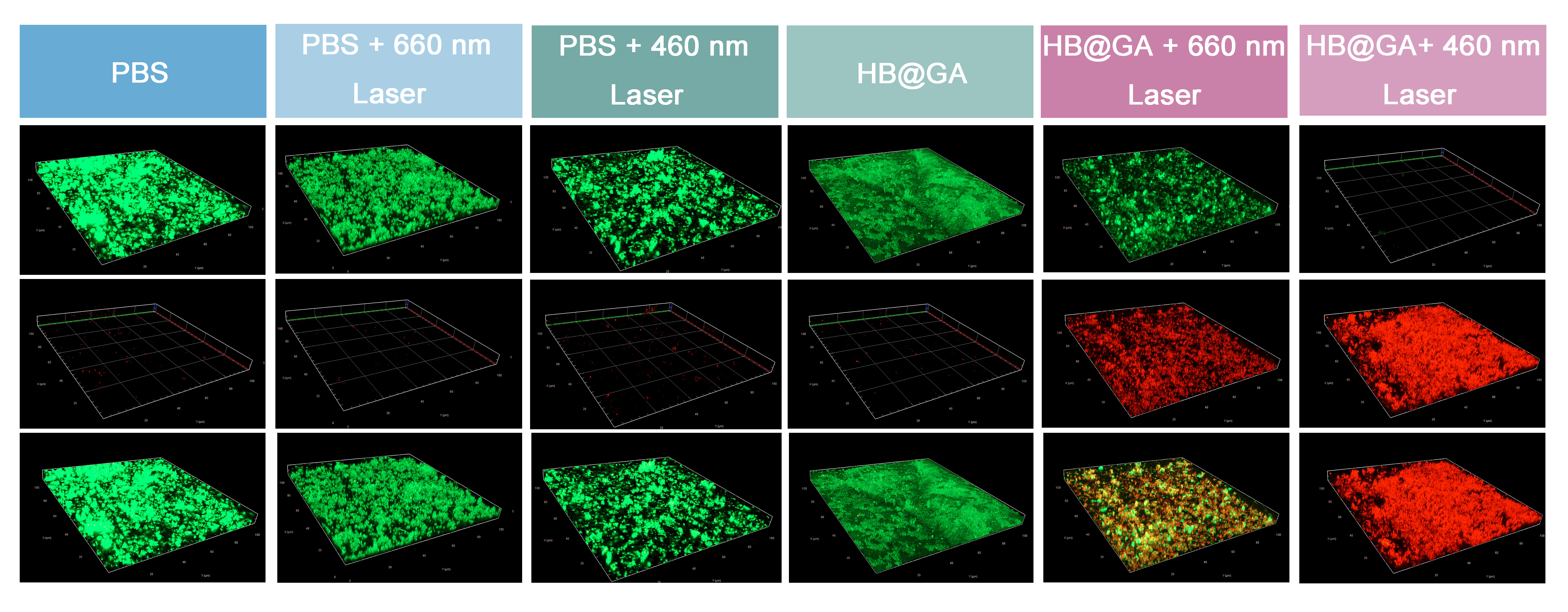


**Figure S7** Live/dead staining of biofilms after different treatments.


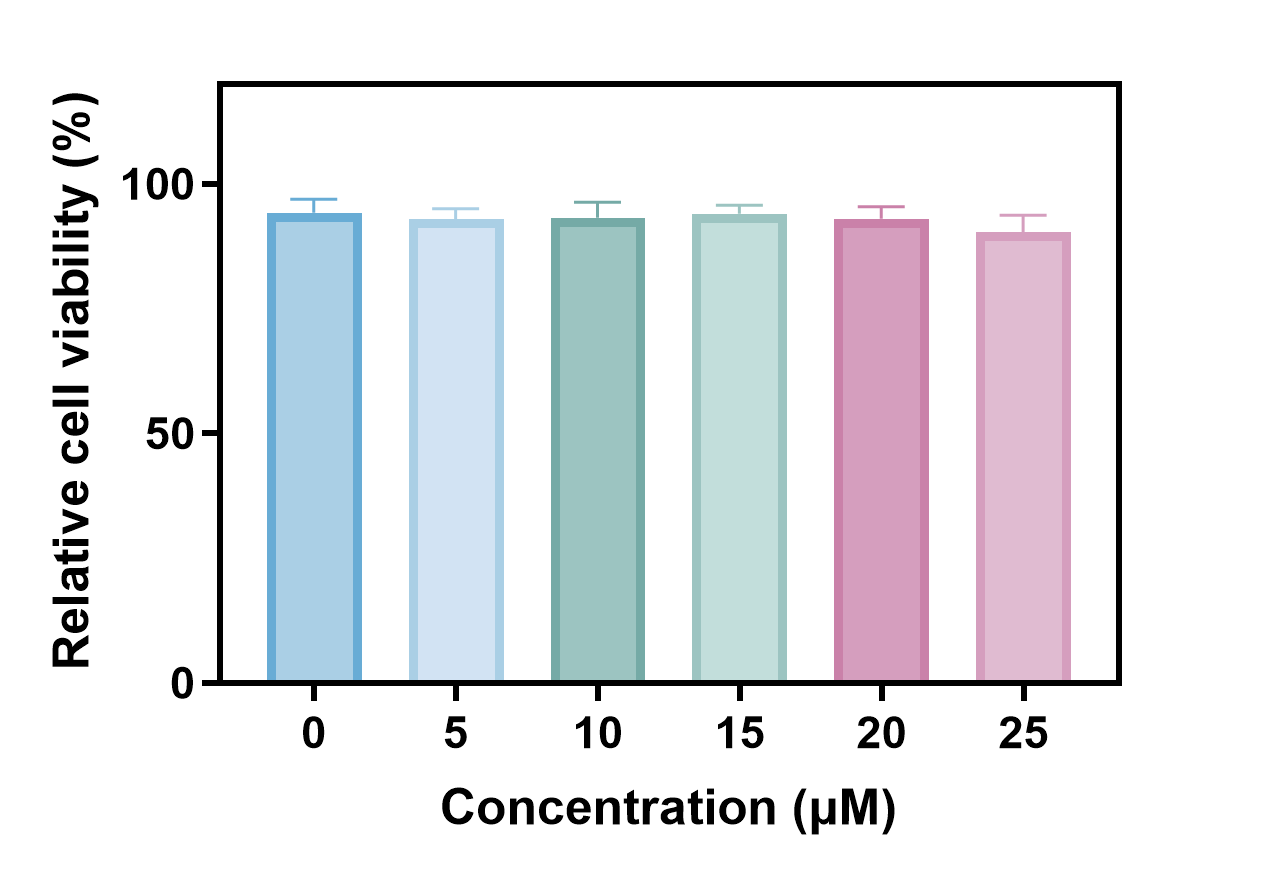


**Figure S8** Cell viability assessed by CCK-8 assay.


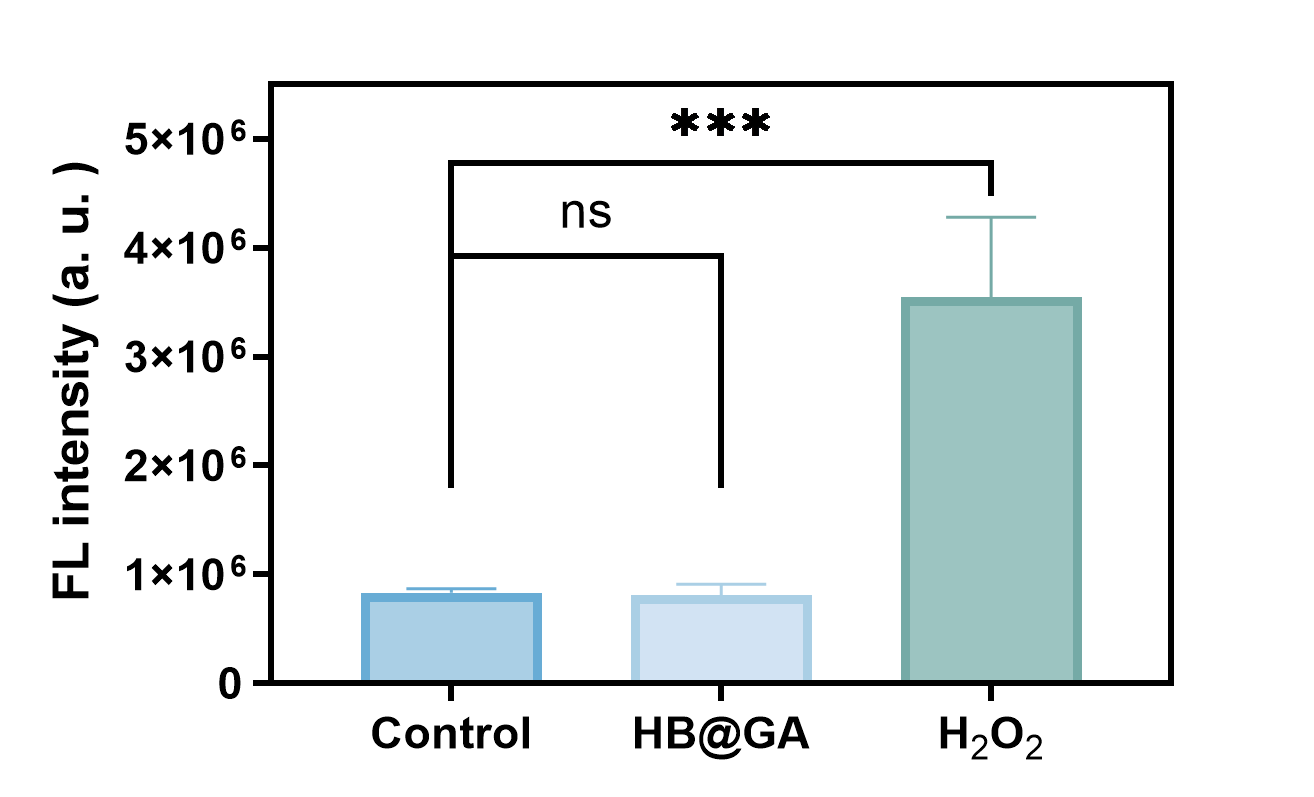


**Figure S9** Intracellular ROS detection in cells.


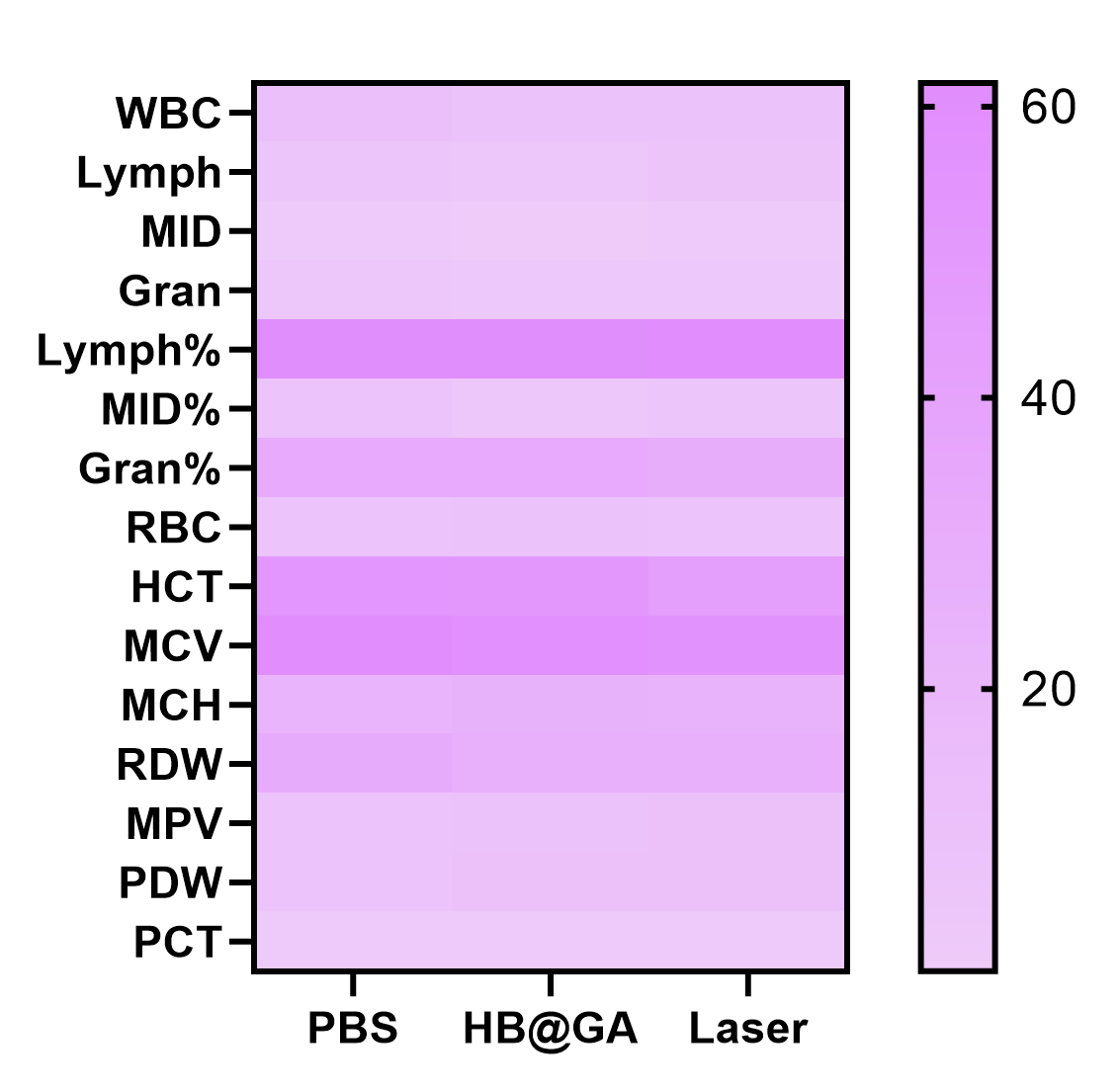


**Figure S10** Blood routine examination of the PBS, HB@GA and Laser group.


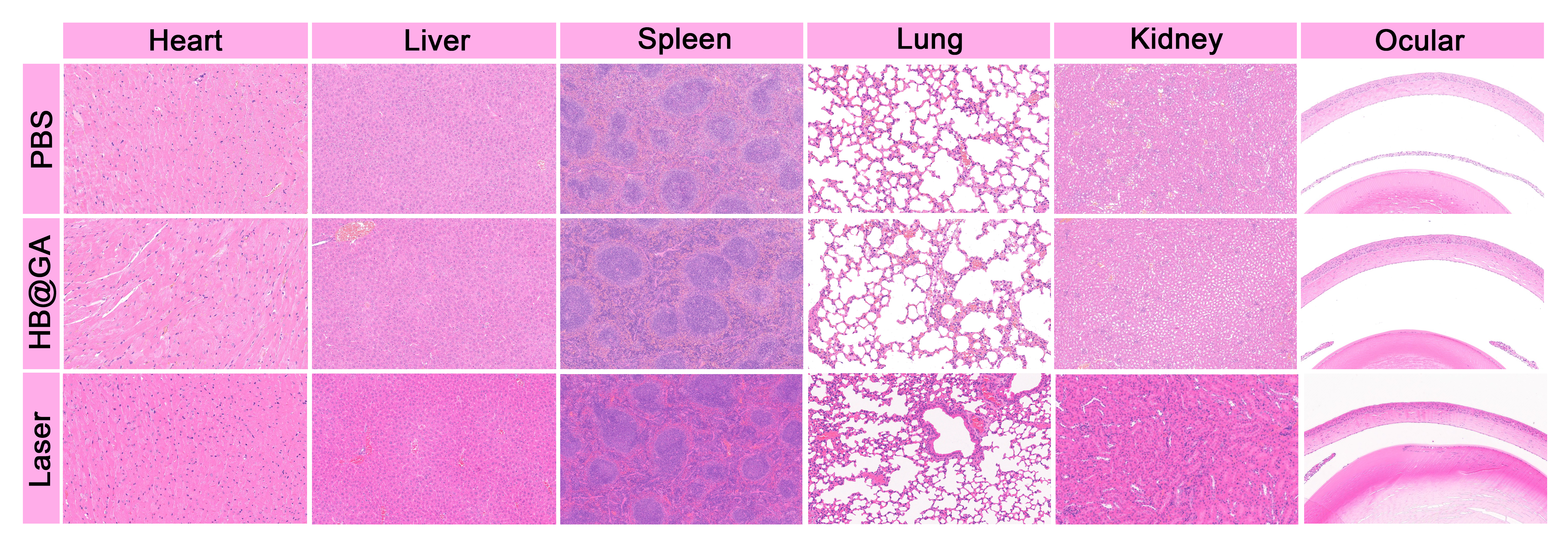


**Figure S11** Histopathologically analysis of major organs of the PBS, HB@GA and Laser group, including the heart, lung, liver, kidney, spleen, and ocular.


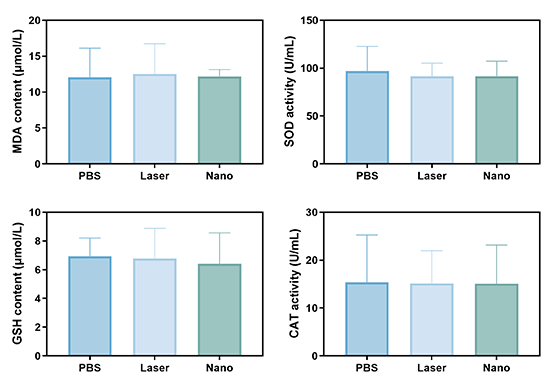


**Figure S12** Detection of oxidative stress markers MDA, SOD, GSH, and CAT.


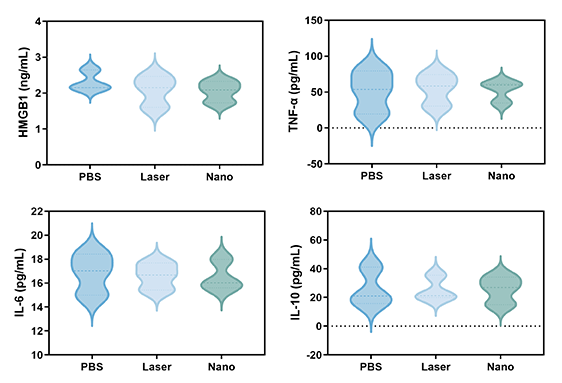


**Figure S13** Detection of inflammatory cytokines HMGB1, TNF α, IL 6, and IL 10.
